# Factors that affect the provision of medical abortion services in Australian primary care: a mixed methods systematic review

**DOI:** 10.5694/mja2.52707

**Published:** 2025-06-18

**Authors:** Greta Skahill, Mridula Shankar

**Affiliations:** ^1^ The University of Melbourne Melbourne VIC; ^2^ Nossal Institute for Global Health, the University of Melbourne Melbourne VIC

**Keywords:** Women’s health, Health services, Abortion, induced, Primary care, Rural health services, Qualitative research

## Abstract

**Objectives:**

To synthesise primary research findings about factors that affect medical abortion provision by general practitioners, nurses, midwives, and pharmacists in Australia.

**Study design:**

Mixed methods systematic review of peer‐reviewed primary publications of qualitative, quantitative, and mixed methods studies of the provision of medical abortion in Australian primary care, 1 January 2013 – 18 January 2025.

**Data sources:**

MEDLINE, Scopus, Web of Science, CINAHL (Cumulative Index to Nursing and Allied Health Literature).

**Data synthesis:**

Twenty‐three publications satisfied our inclusion criteria. We undertook a thematic synthesis of the qualitative study findings to identify barriers and facilitators of medical abortion provision, and assessed the confidence of each review finding using the GRADE‐CERQual approach; we also compared the qualitative synthesis with quantitative study findings. We developed ten review findings grouped under three themes: moral, legal, and regulatory influences on abortion care (three review findings; very low to moderate confidence); the absence of a systems‐based approach to abortion provision (six review findings; moderate to high confidence); and early medical abortion belongs in primary care (one review finding; high confidence). Barriers to providing medical abortion include the absence of a supportive service delivery strategy, insufficient Medicare remuneration, geographic isolation, limited access to training, and colleagues who conscientiously object to abortion. Facilitators of its provision include clinician support networks and personal motivation to improve access to reproductive health care.

**Conclusions:**

A range of individual, service level, and system factors exacerbate the effects of geographic location and financial considerations on the provision of medical abortion in Australian primary care. Our findings indicate that financial and structural support is needed for the geographic decentralisation of medical abortion training and services, the establishment of nurse‐led models of care, and the integration of abortion care into primary care.

Induced abortion is essential health care and access to safe, affordable, high quality abortion care is a human right.^1^ In Australia, abortion legal reforms since 2000 have moved abortion from criminal to health law,^2^ closely aligned with human rights standards and best clinical practice.^3^ Subsidisation of mifepristone by the Pharmaceutical Benefits Scheme in 2013^4^ established early medical abortion (at up to 63 days’ gestation) as a viable option in primary care.^5^ Increasing numbers of prescriptions of the combined mifepristone–misoprostol regimen (MS‐2 Step) indicate that use of this option is growing.^6^ Medical abortion rates are almost twice as high in regional and remote areas as in major cities,^7^ suggesting its value for overcoming access barriers related to geographic location.

The extension of abortion services from specialist clinics to primary care is vital for early medical abortion.^8^ Compared with aspiration techniques, the relative ease of medication administration has spurred telehealth models,^9^, ^10^ recently supported by Medicare rebates.^11^ But medical abortion is not widely offered in primary care^7^ and geographic and financial differences in timely access persist.^12^, ^13^, ^14^ These problems are linked with structural impediments, including regulations regarding the gestational threshold for medical abortion, inadequate public funding, and the absence of a coordinated health systems approach to supporting universal access.^14^, ^15^, ^16^

Centring the provision of abortion within primary care and supportive regulatory and policy environments are crucial to achieving universal access, according to the World Health Organization (WHO).^17^ In Australia, some regulatory barriers that stymied expansion of medical abortion in primary care have been removed; for instance, in August 2023 the Therapeutic Goods Administration (TGA) removed the requirement that all general practitioners and pharmacists who prescribe or dispense MS‐2 Step complete mandatory online training.^18^ Prior to August 2023, only 7% of general practitioners and 22% of pharmacists were active MS‐2 Step providers,^8^, ^19^, ^20^ and most were in major cities.^7^ Other problems include general practitioners’ fears of procedural complications, the stigmatisation of abortion, and insufficient remuneration for prescribing MS‐2 Step.^21^ The introduction of nurse‐ and midwife‐led models of medical abortion has been slow, despite recommendations by the WHO.^17^ The legality of prescribing by nurse practitioners and midwifes is subject to individual state and territory legislation.^22^

All Australian jurisdictions have decriminalised abortion.^3^ The aim of our review is to synthesise primary research findings about factors that affect medical abortion provision by general practitioners, nurses, midwives, and pharmacists in Australia.

## Methods

We report our systematic review according to the Preferred Reporting Items for Systematic Reviews and Meta‐Analyses (PRISMA)^23^ guidelines and the Enhancing Transparency in Reporting the Synthesis of Qualitative Research (ENTREQ)^24^ statement. The protocol was registered with the Open Science Foundation (1 July 2024; https://osf.io/9zqg2).

### Study inclusion criteria

We included primary peer‐reviewed publications on qualitative, quantitative, and mixed methods research on medical abortion provision in Australian primary care published during 1 January 2013 – 18 January 2025; MS‐2 Step was registered by the TGA in August 2012.^4^ We defined provision broadly to include abortion experts, general practitioners, nurses, midwives, and pharmacists. We did not include secondary sources, such as editorials, letters, and commentaries (Box 1).

Box 1 **Factors that affect the provision of medical abortion services in Australian primary care: inclusion and exclusion criteria for our systematic review**

**Table:** 

Criterion	Inclusion	Exclusion
Population	Primary care practitioners: general practitioners, sexual health physicians, nurses, nurse‐practitioners, midwives, pharmacists. Clinical and non‐clinical abortion experts.	Related exclusively to non‐primary care specialists; eg, obstetricians, gynaecologists, accredited and unaccredited registrars.
Intervention	Provision of medical abortion care or its components, including dispensing of MS‐2 Step in Australian primary care. Nurse‐led models of medical abortion care.	Related exclusively to surgical abortion provision. Related exclusively to non‐primary care; eg, tertiary hospitals. Exclusively outside Australia.
Comparison	None.	None.
Outcomes	Barriers to and facilitators of medical abortion provision, including in nurse‐led models.	Related exclusively to abortion access, not provision.
Study design	Primary peer‐reviewed qualitative, quantitative, or mixed methods research. Written in English.	Secondary sources; eg, editorials, letters to the editor, commentaries. Conference abstracts and research protocols.
Time period	1 January 2013 – 18 January 2025.	—

### Search methods

We searched the MEDLINE, Scopus, Web of Science, and CINAHL (Cumulative Index to Nursing and Allied Health Literature) databases for relevant publications. In consultation with a librarian, we developed the primary search strategy in MEDLINE and adapted it for the other databases. The search terms were related to “abortion”, “primary care provision”, and “Australia” (Supporting Information, part 1).

### Study selection

We screened records using Covidence (www.covidence.org). Authors GS and MS screened the titles and abstracts of all articles, then their full text. We resolved discrepancies in screening decisions by discussion. We searched reference lists of included publications to identify further publications, which then underwent the same screening process.

### Data extraction

Using a standardised form, author GS extracted data from each publication, including geographic location (state or territory), study aims, methodological design, participant characteristics (gender, profession, remoteness category, provision status), sample sizes, and study findings (author‐generated themes and explanations, participant quotes, descriptive findings and relationships between independent and dependent variables). Author MS verified the extracted data.

### Assessment of methodological limitations

Authors GS and MS independently appraised all studies and reached a final rating through discussion. We critically appraised the included studies with the Mixed Methods Appraisal Tool (MMAT).^25^ We critically appraised studies that used the Delphi methodology^26^ using the Guidance on Conducting and Reporting Delphi Studies (CREDES) tool.^27^ We did not use quality assessments to exclude studies, but these assessments contributed to assessing confidence in the review findings.

### Data analysis and synthesis

We used thematic synthesis to develop findings from the qualitative data.^28^ Authors GS and MS independently developed line‐by‐line codes for six data‐rich records using NVivo 14. We refined codes to ensure consistency in meaning. GS coded the remaining studies, developing new codes when necessary. We then grouped and summarised codes of similar meanings into broader descriptive categories and discussed their impact on abortion provision to identify barriers and facilitators. Reviewers located these barriers and facilitators in the health care system structure and contextualised them further by gender and rurality. During discussions, we inferred higher order meanings to develop three overarching analytical themes and ten qualitative review findings.

We assessed confidence in each review finding using the GRADE–CERQual approach (https://www.cerqual.org),^29^ which evaluates the confidence of evidence from reviews of qualitative research according to four components: methodological limitations,^30^ how closely the review findings reflect the raw data (coherence),^31^ how applicable the raw data are to the review question (relevance),^32^ and the richness of data supporting the review finding (adequacy).^33^ We assessed the overall confidence (high, moderate, low, very low) in each review finding on component ratings of supporting evidence.

We mapped the quantitative evidence to our qualitative review findings to determine whether the quantitative data supported, extended (ie, added new detail) or contradicted the findings from our qualitative evidence synthesis.

An author reflexivity statement is included in the Supporting Information, part 2.

## Results

A total of 1094 articles were identified in our database and reference list searches; after excluding 899 duplicates and 127 articles deemed not relevant after screening their titles and abstracts, the full text of 68 publications was screened. After excluding a further 45 items, 23 publications were included in our review (Box 2, Box 3; Supporting Information, part 3).^34^, ^35^, ^36^, ^37^, ^38^, ^39^, ^40^, ^41^, ^42^, ^43^, ^44^, ^45^, ^46^, ^47^, ^48^, ^49^, ^50^, ^51^, ^52^, ^53^, ^54^, ^55^, ^56^

Box 2 **Factors affecting the provision of medical abortion services in Australian primary care: a systematic review**

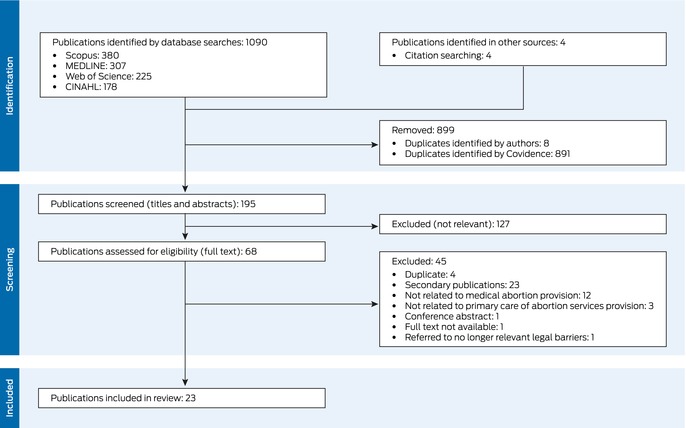

Box 3 **Characteristics of the 23 publications included in our systematic review of publications of studies examining the provision of medical abortion in Australian primary care**

**Table:** 

Characteristic	Number
Study design	
Quantitative	4
Qualitative	14
Mixed methods	3
Delphi method	2
Location	
Victoria	10
New South Wales	3
Queensland	1
Tasmania	1
Australia‐wide	8
Participant remoteness categories*	
Metropolitan areas	14
Regional areas	13
Rural areas	14
Unknown	5
Participants	
General practitioners only	6
General practitioners and nurses only	3
Nurses and midwives only	2
Pharmacists only	1
General practitioners, nurses, pharmacists only	1
Other clinical and non‐clinical experts^†^	10
Medical abortion provision status	
Providers only	9
Providers and others	8
Unknown	6

* Multiple categories for individual studies possible.

† Includes sexual health physicians, service managers, abortion training providers.

### Description of studies

The included publications reported used qualitative (fourteen),^34^, ^35^, ^36^, ^37^, ^38^, ^42^, ^43^, ^46^, ^48^, ^50^, ^51^, ^52^, ^54^, ^55^ quantitative (four),^39^, ^45^, ^47^, ^49^ and mixed methods studies (three);^41^, ^53^, ^56^ one study used the Delphi technique.^40^, ^44^ Ten studies were conducted in Victoria, eight across Australia, three in New South Wales, and one each in Queensland and Tasmania. Fourteen studies provided data on medical abortion provision in rural areas.^37^, ^38^, ^39^, ^40^, ^41^, ^43^, ^44^, ^45^, ^47^, ^48^, ^49^, ^50^, ^52^, ^54^ Eleven studies reported the perspectives of general practitioners or primary care nurses,^28^ ^‐^ ^32^ ^,^ ^35^ ^,^ ^38^ ^‐^ ^40^ ^,^ ^44^ ^,^ ^47^ ^,^ ^37^ ^,^ ^39^ ^,^ ^41^ ^,^ ^43^ ^,^ ^45^ ^‐^ ^51^ three studies those of pharmacists,^34^, ^52^, ^56^ and ten studies reported the views of abortion professionals, including rural abortion training providers^38^ and multidisciplinary abortion experts.^35^, ^36^, ^40^, ^42^, ^44^, ^52^, ^53^, ^54^, ^55^ Seventeen studies reported the views of medical abortion providers.^34^, ^35^, ^36^, ^37^, ^39^, ^40^, ^42^, ^43^, ^44^, ^47^, ^49^, ^50^, ^51^, ^52^, ^53^, ^54^, ^56^

### Methodological limitations

The most frequent methodological limitations of the qualitative studies were related to analytical rigor (partially adequate or unclear in four studies), ethical considerations (partially remediated in six studies) and researcher reflexivity (not discussed in eight studies). For the quantitative studies, the major limitations were sample representativeness (in three of four studies, the samples were only partially representative of the population) and risk of non‐response bias (high or unclear in four studies). The limitations of the mixed methods studies pertained to integrating qualitative and quantitative components. The Delphi study had limitations with regard to defining consensus, participant recruitment, and coherence in design (Box 4; Supporting Information, part 4).

### Themes and findings from the qualitative and quantitative study findings synthesis

The qualitative (Supporting Information, part 5) and quantitative study findings (Supporting Information, part 6) synthesis yielded three overarching themes and ten review findings (Box 4; Box 5):

- moral, legal, and regulatory influences on abortion care (three review findings);
- the absence of a systems‐based approach to abortion provision (six review findings); and
- early medical abortion belongs in primary care (one review finding).

We rated the confidence level for six of our review findings as high, for three as moderate, and for one as very low (Supporting Information, part 5).

Box 4 **Systematic review of the provision of medical abortion services in Australian primary care: summary of qualitative review themes and findings, and assessment of confidence in each review finding (GRADE–CERQual ^29^ )**

**Table:** 

Themes and findings	Overall assessment	Explanation of overall assessment
**Theme** **1. Moral, legal, and regulatory influences on abortion care**		
1. Conscientious objection causes barriers to abortion care at the individual, service, and system levels^34^, ^36^, ^37^, ^41^, ^42^, ^43^, ^44^, ^46^, ^48^, ^50^, ^54^, ^56^	Moderate confidence	Methodological limitations: moderate concerns (recruitment, data collection, analytical rigor, coherence of design, reflexivity, rationale for mixed methods approach, integration and interpretation of qualitative and quantitative components). Coherence: no or very minor concerns. Relevance: moderate concerns. Adequacy: No or very minor concerns (twelve studies with moderately thick data).*
2. Decriminalisation is crucial but insufficient for expanding abortion care^36^	Very low confidence	Methodological limitations: no or very minor concerns. Coherence: no or very minor concerns. Relevance: serious concerns (sole article only indirectly relevant to review). Adequacy: serious concerns (one article with relatively thick data).
3. Establishing an autonomous nurse‐led model of medical abortion requires regulatory reform and overcoming health system barriers^35^, ^44^, ^48^, ^52^, ^53^, ^54^, ^55^, ^56^	Moderate confidence	Methodological limitations: moderate concerns (process, recruitment, analytical rigor, coherence of design, link from data to findings, reflexivity, rationale for mixed methods approach, integration and interpretation of qualitative and quantitative components). Coherence: no or very minor concerns. Relevance: minor concerns. Adequacy: no or very minor concerns on adequacy (eight articles with moderately thick data).
**Theme 2. The absence of a systems‐based approach to abortion provision**		
4. Primary and ancillary providers of medical abortion are not well connected^35^, ^37^, ^38^, ^41^, ^43^, ^44^, ^48^, ^50^, ^52^, ^54^, ^55^, ^56^	High confidence	Methodological limitations: minor concerns (recruitment, data collection, analytical rigor, coherence of design, link from data to findings, reflexivity, rationale for mixed methods approach, integration and interpretation of qualitative and quantitative components). Coherence: no or very minor concerns. Relevance: minor concerns. Adequacy: no or very minor concerns on adequacy (twelve articles with moderately thick data).
5. Preparedness and value ascribed to training, qualifications, and clinical experience^34^, ^35^, ^37^, ^48^, ^50^, ^51^, ^52^, ^54^, ^55^, ^56^	High confidence	Methodological limitations: minor concerns (coherence of design, analytical rigor, link from data to findings, rationale for mixed methods approach, integration and interpretation of qualitative and quantitative components). Coherence: no or very minor concerns. Relevance: minor concerns. Adequacy: minor concerns (ten articles with relatively thin data).
6. The absence of a visible service system and a culture of secrecy obscure levels of abortion demand^34^, ^36^, ^37^, ^41^, ^43^, ^46^, ^54^	Moderate confidence	Methodological limitations: moderate concerns (recruitment, data collection, coherence of design, analytical rigor, reflexivity, rationale for mixed methods approach, integration of qualitative and quantitative components). Coherence: no or very minor concerns. Relevance: minor concerns. Adequacy: minor concerns (seven articles with relatively thin data).
7. Inadequate resources and geographic isolation are barriers to rural abortion care^35^, ^37^, ^38^, ^41^, ^42^, ^44^, ^48^, ^50^, ^54^, ^55^	High confidence	Methodological limitations: minor concerns (recruitment, data collection, coherence of design, analytical rigor, reflexivity, rationale for mixed methods approach, integration of qualitative and quantitative components). Coherence: no or very minor concerns. Relevance: minor concerns. Adequacy: no or very minor concerns (ten articles with moderately thin data).
8. Financial disincentives and the gendered nature of abortion care contribute to work overload, service fragmentation, and gender‐based pay disparities^36^, ^37^, ^38^, ^43^, ^44^, ^46^, ^48^, ^51^, ^52^, ^54^, ^55^	High confidence	Methodological limitations: minor concerns (process, recruitment, link from data to findings, coherence of designs). Coherence: no or very minor concerns. Relevance: minor concerns. Adequacy: no or very minor concerns (eleven articles with relatively thin data).
9. Anticipated and actual stigmatisation of abortion affect its provision^36^, ^37^, ^43^, ^46^, ^54^, ^55^	High confidence	Methodological limitations: minor concerns (process, recruitment, link from data to findings, coherence of designs, analytical rigor, ethics, reflexivity). Coherence: no or very minor concerns. Relevance: minor concerns. Adequacy: minor concerns (seven articles with relatively thin data).
**Theme 3. Early medical abortion belongs in primary care**		
10. Medical abortion in primary care enhances equity and patient autonomy^35^, ^37^, ^38^, ^43^, ^48^, ^50^, ^51^, ^52^, ^54^	High confidence	Methodological limitations: minor concerns (coherence of designs, analytical rigor, ethics, reflexivity). Coherence: no or very minor concerns. Relevance: no or very minor concerns. Adequacy: no or very minor concerns (nine articles with relatively thick data).

* Adequacy assessment evaluates both the quantity and richness of data contributing to a review finding. Reviewers gauge data richness by appraising the depth of information, rating excerpts from very thin to very thick based on their explanatory detail. The overall adequacy judgement combines this assessment of data richness across all contributory studies with the total data quantity.^33^

Box 5 Map of review findings by article and publication year*

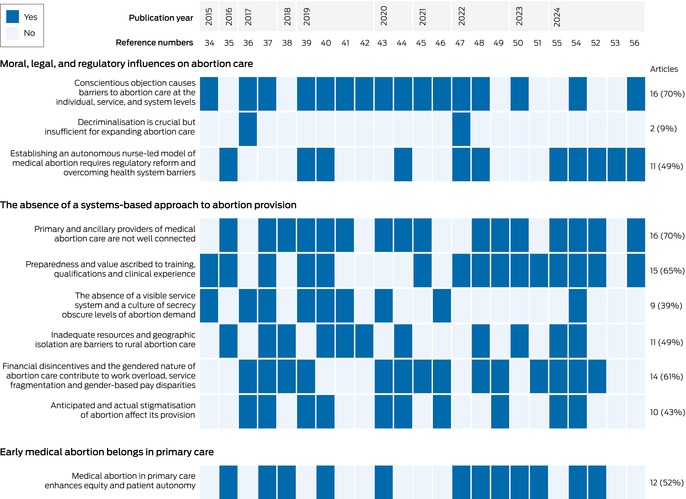

* Format adapted from figure 2 in reference 57.

#### Moral, legal, and regulatory influences on abortion care

*Finding 1: Conscientious objection causes barriers to abortion care at the individual, service, and system levels* (moderate confidence). Moral or religious beliefs are an individual barrier to providing abortion care for some doctors and pharmacists; colleagues who conscientiously object to abortion care greatly limit its provision and clinical training. In such cases, the service (including the dispensing of MS‐2 Step) is not offered, care is delayed, or providers must offer care clandestinely. Health services use conscientious objection legal clauses to justify institutional bans on abortion provision and education.^34^, ^36^, ^37^, ^41^, ^42^, ^43^, ^44^, ^46^, ^48^, ^50^, ^54^, ^56^

In Victoria, section 8 of the *Abortion Law Reform Act* 2008 (the legal clause permitting conscientious objection) is perceived by some general practitioners as a mechanism for facilitating abortion access via referral.^43^ However, abortion providers are concerned that section 8 legitimises refusing to provide care without adequate justification, and that it is routinely misused by pharmacists and general practitioners who do not fulfil professional obligations and legal requirements to facilitate continuity of care by referring women seeking abortion.^36^, ^37^, ^42^, ^43^

Quantitative studies found that some primary care clinicians conscientiously object to abortion,^39^, ^41^, ^45^, ^47^ but personal opposition to abortion is not always a barrier to providing it.^47^ Conscientious objection seems to be more frequent among general practitioners trained overseas,^41^ and increases with time since qualification for registered nurses and midwives.^47^ Conscientious objection by colleagues, practice‐wide bans on abortion, and pharmacist refusal to dispense MS‐2 Step limit the provision of abortion provision by general practitioners and primary care nurses.^39^, ^40^

*Finding 2: Decriminalisation is crucial but insufficient for expanding abortion care* (very low confidence). In Victoria, decriminalisation of abortion was viewed by providers as important for indicating that abortion care is health care. It was also understood by some as a legal mechanism for reducing unequal decisional dynamics for abortion seekers and providers of abortion care. However, in the absence of government support for service provision, including by establishing a sustainable health workforce, decriminalisation alone was considered insufficient for expanding services.^36^

This finding was supported by the findings of a quantitative study in Queensland which suggest that decriminalisation of abortion did not significantly alter support for the public provision of abortion care among sexual health nurses and midwives.^47^

*Finding 3: Establishing an autonomous nurse‐led model of medical abortion requires regulatory reform and overcoming health system barriers* (moderate confidence). Regulations prohibiting nurse practitioners prescribing MS‐2 Step, Medicare billing requirements for general practitioner involvement, and scarce training opportunities are systemic barriers that limit the autonomous provision of medical abortion by primary care nurses. At the service level, practice nurse involvement in medical abortion depends on their employers’ interest and approval, as well as clear protocols for task sharing. Conscientious objection by colleagues in regional and rural organisations can limit nurse involvement, although some organisations have adopted alternative approaches, including task sharing with telehealth providers. Primary care nurses have communication skills suited for abortion care, and their involvement eases the general practitioner workload. However, some may not have the physical or psychological capacity to independently provide abortions and manage complications without appropriate training, provide after‐hours support, or achieve wider medical community endorsement.^35^, ^44^, ^48^, ^52^, ^53^, ^54^, ^55^, ^56^

Conflicting views among abortion experts about whether primary care nurses can manage abortion care and complications independently is a barrier to developing nurse‐led models. While abortion providers generally endorse the involvement of practice nurses, they see their role as being supportive rather than independent.^35^, ^44^, ^52^, ^55^

Quantitative findings also suggest that differing views on the nursing and midwifery scope of practice, concerns about managing complications, limited abortion training opportunities, and the emotional demands of abortion work are all barriers to nurse involvement in medical abortion care.^39^, ^47^, ^53^ Abortion experts agree that nurse‐led models are needed to expand access to abortion, but have conflicting views about the required degree of general practitioner involvement.^40^, ^53^ The financial and logistical feasibility of nurse‐led models requires extensive government and primary health care network support, as well as endorsement by peak nursing bodies.^40^

#### The absence of a systems‐based approach to abortion provision

*Finding 4: Primary and ancillary providers of medical abortion are not well connected* (high confidence). Comprehensive support by a network of ancillary services (general practitioners, pharmacists, sonographers, psychologists, referral hospitals) is essential for providing high quality abortion care. Establishing such a network, including guaranteeing the support of local hospitals for emergency and after‐hours care, is logistically difficult for general practitioners, as some services obstruct or refuse to support medical abortion provision.^35^, ^37^, ^38^, ^41^, ^43^, ^44^, ^48^, ^50^, ^52^, ^54^, ^55^, ^56^

In the absence of systemwide support, local and virtual peer support networks and phone helplines enhance clinician knowledge, confidence, and resources, and reduce feelings of isolation.^35^, ^38^, ^43^, ^50^, ^52^, ^54^, ^56^ Such networks are especially useful for decentralised abortion services in rural areas.^38^, ^54^ The MS‐2 Step 24‐hour nurse hotline was perceived to be helpful for after‐hours support and more accessible than assistance from local hospitals.^43^, ^52^ In Victoria, 1800 My Options, a service for information on sexual and reproductive health care, is a vital resource for providers.^52^

Quantitative studies found that inadequate access to ancillary services (pathology and ultrasound), abortion medications, and tertiary support for complications are barriers to providing medical abortion.^39^, ^40^, ^45^ Primary care nurses are particularly concerned about access to surgical support.^39^ Service delivery models that encompass ancillary providers and provide clear referral pathways are regarded as the most important factor in service expansion.^40^, ^49^

*Finding 5: Preparedness and value ascribed to training, qualifications, and clinical experience* (high confidence). Insufficient knowledge, training, and abortion care experience are barriers to in‐person and telehealth abortion provision for primary care clinicians. Many providers pursue external training or qualifications to compensate the limited opportunities provided by their workplaces, medical curricula, or clinical placements. Access to supervision and hands‐on learning improves general practitioners’ skills and confidence, as exposure to medical abortion in primary care is often sporadic. Some experienced providers feel that medical abortion should be provided in specialist clinics where skills and experiences in women's health are stronger and demand for the service is consistently higher.^34^, ^35^, ^37^, ^48^, ^50^, ^51^, ^52^, ^54^, ^55^, ^56^

Quantitative studies also found that lack of knowledge,^39^, ^40^, ^45^, ^49^, ^56^ training opportunities^39^, ^45^ and guidelines^49^ reduce clinician preparedness. Prior experience in providing abortion care is valued more by general practitioners than by primary care nurses.^49^ Primary care providers would like to receive abortion training,^45^, ^47^ including as part of the core curriculum.^47^

*Finding 6: The absence of a visible service system and a culture of secrecy obscure levels of abortion demand* (moderate confidence). The absence of a visible primary care service system for abortion, particularly in rural areas, means referrers rely on “rumours” to identify providers offering abortion care. The stigmatisation of abortion and privacy concerns cause practitioners to operate by stealth, leading to conflicting perceptions of demand: some fear being overwhelmed, others regard demand as low. Abortion experts worry that without state or federal government support for abortion care, service expansion and workforce development will not increase.^34^, ^36^, ^37^, ^41^, ^43^, ^46^, ^54^

Quantitative studies found that the awareness of clinicians in rural areas of local abortion services is poor and believe that demand is limited by privacy concerns.^41^ A small minority believe that the available abortion services are adequate.^39^, ^41^ Increasing public awareness of the availability of medical abortion in primary care could increase the demand for local abortion care, which could increase its provision.^40^

*Finding 7: Inadequate resources and geographic isolation are barriers to rural abortion care* (high confidence). General practitioners in rural areas who offer abortion care feel isolated, anticipate stigmatisation, and experience pressure and emotional distress, especially when working in towns or areas with conscientious objectors. Funding models do not consider these problems, and some rural providers do not have adequate resources or financial compensation to meet community needs, leading to staff burnout, poor workforce retention, and reduced quality of care (eg, delayed appointments).^35^, ^37^, ^38^, ^41^, ^42^, ^44^, ^48^, ^50^, ^54^, ^55^

Geographic decentralisation of rural medical abortion services relies on the formation of partnerships with locally embedded sexual health organisations or intermediaries who facilitate training, protocol, and service development.^35^, ^38^ Leveraging of telehealth and general practitioner and nurse task‐sharing approaches have helped overcome obstacles associated with conscientious objections.^54^

Quantitative studies have found that rural providers of medical abortion do not have access to ultrasound, allied health, surgical, and after‐hours support. Expanding rural medical abortion services requires more resources and government support to provide incentives for training and professional development.^40^

*Finding 8: Financial disincentives and the gendered nature of abortion care contribute to work overload, service fragmentation, and gender‐based pay disparities* (high confidence). The fragmented structure of public health care financing and reliance on time‐based Medicare item numbers discourage practitioners from providing abortion care. In the absence of a publicly funded supportive framework for integrating abortion services into primary care, including using telehealth, individual providers must navigate several logistical hurdles at their own cost. Coupled with the gendered nature of abortion provision and female providers often working part‐time, delays and fragmentation in care contribute to work overload and risk of burn‐out at the individual level, and gender‐related pay disparities and organisational glass ceilings at the service level.^36^, ^37^, ^38^, ^43^, ^44^, ^46^, ^48^, ^51^, ^52^, ^54^, ^55^

Offering person‐centred abortion care requires time and empathic communication that is not financially compensated by the current funding system, leaving providers feeling undervalued.^48^ Time and financial pressures can affect service quality, and some providers feel compelled to prioritise clinical considerations over other aspects, such as cultural safety.^48^, ^51^

Quantitative studies have found that financial barriers to medical abortion provision include the time required for abortion counselling, legal restrictions of autonomous nurse provision,^49^ and financial unviability of the service.^49^ Financial considerations were more frequently a significant barrier for male than female general practitioners.^45^

*Finding 9: Anticipated and actual stigmatisation of abortion affect its provision* (high confidence). Anticipating that medical abortion provision will detrimentally affect or otherwise reduce one's professional reputation and practice is an internal barrier for general practitioners, and rural providers fear negative feedback from their communities. Some providers experience moral disapproval by friends and colleagues and choose not to advertise their service for fear of anti‐abortion activists.^36^, ^37^, ^43^, ^46^, ^54^, ^55^

Some practitioners who do not provide abortion care do not believe that stigmatisation affects cases in which the patient and provider do not have negative cultural or religious beliefs about abortion, and in workplaces where women's reproductive health is a priority.^37^

Quantitative studies have found that primary care clinicians, particularly nurses, are concerned about being known as abortion providers because they anticipate stigmatisation by colleagues, friends, and their community,^39^, ^40^, ^49^ and fear harassment by anti‐abortion activists.^39^, ^40^

### Early medical abortion belongs in primary care

*Finding 10: Medical abortion in primary care enhances equity and patient autonomy* (high confidence). General practitioner providers are motivated by the belief that abortion care is integral to women's health care and should be financially, geographically, and socially accessible. This sense is greater among clinicians who provide care to socially marginalised, disadvantaged, or rural women for whom access to services may not be straightforward. Medical abortion provision in primary care provides greater continuity of care (eg, for follow‐up and contraception) and facilitates the tailoring of care to the needs of the woman and the community, including with telehealth.^35^, ^37^, ^38^, ^43^, ^48^, ^50^, ^51^, ^52^, ^54^

The risk of inadequate follow‐up is worrying for practitioners who provide or do not provide abortion care and can lead to their no longer providing medical abortion.^37^, ^43^ This concern appears greatest for general practitioners who provide a low cost services to women from outside their local area.^37^ Provider acceptance of telehealth has grown, but barriers to uptake include difficulties with building rapport, lack of control over patients’ physical surroundings, and the inability to undertake physical examinations.^52^

Quantitative studies have found that recognition of abortion care as health care and the need to increase access in marginalised communities are important factors for increasing its provision by general practitioners and primary care nurses,^40^, ^47^, ^49^ but fear of loss to follow‐up is a concern.^49^

## Discussion

Our review synthesises the findings of studies of the barriers and facilitators of medical abortion provision in Australian primary care. At the systems level, key barriers include the absence of a clear service delivery strategy, insufficient Medicare remuneration, and limited training opportunities and ancillary support. Service level barriers include resource constraints, geographic isolation, and working with conscientious objectors to abortion. Individual barriers include insufficient abortion knowledge and experience, and personal beliefs regarding abortion. Access to clinician support networks and a commitment to enhancing reproductive health care facilitate its provision.

The strengths of our study include the fact that we optimised the use of data reported by studies with different study designs. Our application of GRADE‐CERQual to each finding enhances the usability of our findings in decision making. We also discussed factors affecting telehealth and nurse‐led models of abortion care.

Our findings suggest that the decision to provide abortion care is based on core personnel values, challenging the popular view that conscientiousness is linked only with opposition to abortion.^58^, ^59^ The values that underpin this conscientiousness include viewing abortion as essential health care, supporting universal access to reproductive choice, and wanting to meet community health needs.^58^, ^59^, ^60^ Values clarification workshops that enhance providers’ self‐awareness and understanding of their professional duty in abortion care is a promising strategy for supporting the Australian primary care workforce.^61^, ^62^ These workshops foster supportive attitudes and reduce active opposition among providers with diverse beliefs and in different contexts.^62^, ^63^ The challenges posed by conscientious objection must prompt consideration of laws that protect the right to decline involvement in pregnancy termination, which allow practitioners to avoid their professional duty to provide essential reproductive health care,^64^ but providing insufficient protection for practitioners who do provide it.

We found that most medical abortion providers are women, and that there are financial disincentives for engaging in sexual and reproductive health work. Recent scrutiny of the Medicare Benefits Scheme (MBS)^65^ affirms the existence of gender‐related biases in federal funding structures; for example, the MBS rebates for women's health procedures are smaller than for men's health procedures. This discrepancy discourages clinicians from engaging in women's health care. Widespread uptake of medical abortion services in general practice is unlikely without redressing these imbalances, and we welcome the recent announcement of a gender‐based audit of the MBS system.^11^

A key obstacle to medical abortion provision is the limited opportunity to gain clinical experience. This barrier is linked with the dearth of formal abortion training in medical schools,^66^ exacerbated by bans on abortion care education in religious training institutions, an example of abortion exceptionalism: beliefs and practices that distinguish abortion from standard medical care because it is immoral, risky, or too specialised, resulting in its systematic exclusion from training curricula.^67^ Integrating abortion services into primary care through a whole of system approach could shift clinical perceptions of what constitutes standard health care.

Our findings reinforce the importance of establishing communities of practice, particularly for rural providers. It has recently been reported that the Australian Contraception and Abortion Primary Care Practitioner Support Network (AusCAPPS) is widely used by general practitioners, nurses, and pharmacists to increase knowledge about abortion and to clarify questions about service provision.^68^ A 2023 Senate enquiry has consequently recommended to continue federal funding of the network.^14^

Our review emphasises the suitability of primary care for providing medical abortion, but nurses and midwives could play greater roles. Recent legislative changes in Queensland,^69^ the Australian Capital Territory,^70^ and Western Australia^71^ permit nurse practitioners and endorsed midwives to prescribe MS‐2 Step. Increasing the involvement of nurses and midwives in medical abortion will also require nurse‐ and midwife‐specific abortion training, improved Medicare remuneration, and overcoming the perception that medical abortion is outside their scope of care. Expanding the evidence base for nurse and midwife‐led models of medical abortion in Australia, including by task sharing,^72^ could change this situation.

### Limitations

Only three studies examined the role of pharmacists,^34^, ^52^, ^56^ limiting our conclusions about factors that affect the dispensing of MS‐2 Step. Some studies also examined surgical abortion or included information about non‐primary care providers. Most studies were undertaken in Victoria, particularly those that investigated conscientious objection, and there were no studies from Western Australia, South Australia, or the Northern Territory.

### Conclusion

Regulatory, governance, funding, and service coordination barriers need to be overcome to improve early medical abortion delivery in Australian primary care. Such care is important for supporting the National Women's Health Strategy goal of equitable access to abortion care.^73^

## Open access

Open access publishing facilitated by the University of Melbourne, as part of the Wiley – the University of Melbourne agreement via the Council of Australian University Librarians.

## Competing interests

No relevant disclosures.

Received 30 October 2024, accepted 24 February 2025

## Supporting information


Supplementary methods and results
