# Supplementary material for: Factors that affect the provision of medical abortion services in Australian primary care: a mixed methods systematic review
Source: Med J Aust. 2025 Jun 18;223(2):101–10. doi: 10.5694/mja2.52707 (PMC12276729; doi:10.5694/mja2.52707)
Supplement: Supplementary file 1 — Supplementary methods and results [file MJA2-223-101-s001.pdf]

Supporting Information

Supplementary methods and results

This appendix was part of the submitted manuscript and has been peer reviewed.
It is posted as supplied by the authors.

Appendix to: Skahill G, Shankar M. Factors that affect the provision of medical abortion services in Australian primary care: a mixed methods systematic review. *Med J Aust* 2025; doi: 10.5694/mja2.52707.

Supplementary methods

1. Search strategies

Medline

#	Searches
1	exp Abortion, Induced/
2	exp Abortifacient Agents/
3	(medica* adj3 abort*).mp.
4	MTOP.mp.
5	(mifepristone or misoprostol).mp.
6	(induc* adj2 abort*).mp.
7	(pregnancy adj2 termination adj2 medica*).mp.
8	(early adj3 abort*).mp.
9	RU486.mp.
10	1 or 2 or 3 or 4 or 5 or 6 or 7 or 8 or 9
11	exp Primary Health Care/
12	exp General Practice/
13	Family Planning Services/
14	general pract*.mp.
15	nurse pract*.mp.
16	(primary adj1 (health or setting or care or healthcare or services)).mp.
17	(Marie Stopes or Dr Marie).mp.
18	((rural or reproductive or sexual or women's) adj2 (health or care or healthcare or services)).mp.
19	((fellows or specialists) and RANZCOG).mp.
20	(Telehealth or telemedicine or telephone).mp.
21	(pharmacy or pharmaci*).mp.
22	11 or 12 or 13 or 14 or 15 or 16 or 17 or 18 or 19 or 20 or 21
23	exp Australia/
24	(Australia or Australian).mp.
25	(Victoria or Victorian).mp.
26	New South Wales.mp.
27	Queensland.mp.
28	Northern Territory.mp.
29	((Western or South) adj1 Australi*).mp.
30	Tasmani*.mp.
31	23 or 24 or 25 or 26 or 27 or 28 or 29 or 30
32	10 and 22 and 31
33	limit 32 to (english language and yr="2013 -Current")

Web of Science

#	Searches
1	TS=((medica* near/2 abort*) OR mtop OR mifepristone OR misoprostol OR (induc* near/1 abort*) OR (pregnancy near/1 termination near/1 medica*) OR (early near/2 abort*) OR ru486)
2	TS=("General pract*" OR "nurse pract*" OR (primary near/0 (setting OR healthcare OR services OR health OR care)) OR "Marie Stopes" OR "Dr Marie" OR ((rural OR reproductive OR sexual OR womens) near/1 (health OR care OR healthcare OR services)) OR ((fellows OR specialists) AND ranzcog) OR telehealth OR telemedicine OR telephone OR pharmacy OR pharmaci* OR "family planning services")
3	TS=(australia OR australian OR victoria OR victorian OR "New South Wales" OR queensland OR "Northern Territory" OR ((western OR south) near/1 australi*) OR tasmani*)
4	#1 AND #2 AND #3
5	PY=2013-2025
6	#5 AND #4

CINAHL

#	Searches
S1	MH (“Abortion, Induced+" OR "Abortifacient Agents+”)
S2	TI ((medica* n2 abort*) OR MTOP OR mifepristone OR misoprostol OR (induc* n1 abort*) OR (pregnancy n1 termination n1 medica*) OR (early n2 abort*) OR RU486) OR AB ((medica* n2 abort*) OR MTOP OR mifepristone OR misoprostol OR (induc* n1 abort*) OR (pregnancy n1 termination n1 medica*) OR (early n2 abort*) OR RU486)
S3	S3 MH (“Primary Health Care" OR “family practice” OR "Family Planning+”)
S4	TI (primary w0 (care OR health OR healthcare OR services OR setting)) OR AB (primary w0 (care OR health OR healthcare OR services OR setting))
S5	TI (telehealth OR telemedicine OR telephone OR pharmacy OR pharmaci* OR "family planning services”) OR AB (telehealth OR telemedicine OR telephone OR pharmacy OR pharmaci* OR "family planning services”)
S6	TI (“General pract*" OR "nurse pract*" OR "Marie Stopes" OR "Dr Marie") OR AB (“General pract*" OR "nurse pract*" OR "Marie Stopes" OR "Dr Marie")
S7	TI ((fellows OR specialists) AND ranzcog) OR AB ((fellows OR specialists) AND ranzcog)
S8	(MH "Australia+")
S9	TI (Australia OR Australian OR Victoria OR Victorian OR "New South Wales" OR Queensland OR "Northern Territory" OR Tasmani*) OR AB (Australia OR Australian OR Victoria OR Victorian OR "New South Wales" OR Queensland OR "Northern Territory" OR Tasmani*)
S10	TI ((Western OR South) w0 Australi*) OR AB ((Western OR South) w0 Australi*)
S11	S1 OR S2
S12	S3 OR S4 OR S5 OR S6 OR S7
S13	S8 OR S9 OR S10
S14	S11 AND S12 AND S13
Limiters	Published Date: 20130101-20250131

Scopus

Scopus search was based on Web of Science search strategy

2. Review team and reflexivity

The review team authors differ in age, profession and levels of experience in sexual and reproductive healthcare research. GS's relationship with the subject matter is both personal and professional. As a young, cisgender woman and junior doctor working at a Catholic health service which does not provide abortions, GS is keenly aware of her own privilege in being able to navigate the healthcare system to obtain safe and timely care. Having held sexual and reproductive health education roles in the past, GS's data analysis and interpretation of review findings inevitably occurred through an advocacy and pro-choice lens. MS is a post-doctoral researcher with over 15 years of experience conducting research in sexual and reproductive health across diverse geographical settings. Her commitment to conducting research to promote universal access to abortion is deeply rooted in her lived experience as a cisgender Indian woman, which has fostered a strong and innate understanding of the importance of comprehensive reproductive health services, including primary (contraception) and secondary (abortion) pregnancy prevention approaches. She firmly believes that having agency over one's reproductive choices is key to personal freedom and self-determination. These values underpin her research on abortion and her analytical approaches to the data. MS provided methodological guidance to GS. The review authors' similar ideological standpoints on the need for greater abortion care accessibility influenced their decision to conduct this review together and shaped the ways they interpreted the data and synthesised the findings.

Supplementary results

3. Characteristics of the included articles

Author/Year	Title	Stated aims/objectives	Methodology	Geographical location	Participant remoteness	Participants (sample size)	Medical abortion provision status	Participant gender
Lee 2015[34]	Mifepristone (RU486) in Australian pharmacies: the ethical and practical challenges	The objective of this study was to explore attitudes and knowledge of Australian pharmacists about mifepristone/misoprostol	Qualitative (semi-structured interviews)	Sydney, NSW	Unknown	Pharmacists: 41<br>Total: 41	Providers and non-providers	Unknown
Newton 2016[35]^	...a one stop shop in their own community': Medical abortion and the role of general practice	To investigate the potential for expanding the role of general practice in the provision of medical abortion in Victoria	Qualitative (semi-structured interviews)	Victoria	Metropolitan areas, regional areas	General practitioners: 5<br>Obstetrician gynaecologists: 4<br>Medical practitioners: 3<br>Service managers: 3<br>Primary care nurses: 2<br>Psychologist: 1<br>Sexual health physician: 11<br>Total: 19	Providers and non-providers	Male: 4<br>Female: 15
Keogh 2017[36]^	Intended and unintended consequences of abortion law reform: perspectives of abortion experts in Victoria, Australia	To explore how experts in abortion service provision perceive the intent and subsequent impact of the 2008 Victorian abortion law reform on their practice	Qualitative (semi-structured interviews)	Victoria	Metropolitan areas, regional areas	General practitioners: 5<br>Obstetrician gynaecologists: 4<br>Medical practitioners: 3<br>Service managers: 3<br>Primary care nurses: 2<br>Psychologist: 1<br>Sexual health physician: 11<br>Total: 19	Providers only	Male: 4<br>Female: 15
Dawson 2017[37]	Medical termination of pregnancy in general practice in Australia: a descriptive-interpretive qualitative study	To explore the provision and referral of medical termination of pregnancy by general practitioners	Qualitative (semi-structured interviews and single focus group)	New South Wales	Metropolitan areas, regional areas, rural areas	General practitioners: 31<br>General practitioner surgeon: 1<br>Total: 32	Providers and non-providers	Male: 9<br>Female: 24

Author/Year	Title	Stated aims/objectives	Methodology	Geographical location	Participant remoteness	Participants (sample size)	Medical abortion provision status	Participant gender
Hulme-Chambers 2018[38]	Medical termination of pregnancy service delivery in the context of decentralization: social and structural influences	To investigate the factors that enabled and challenged a decentralization effort to increase rural medical termination of pregnancy service provision in Victoria	Qualitative (semi-structured interviews)	Victoria	Metropolitan areas, rural areas	Abortion training providers: 6<br>General practitioners: 7<br>Registered nurses: 6<br>Total: 19	Unknown	Male: 2<br>Female: 17
de Moel-Mandel 2019b[39]	Snapshot of medication abortion provision in the primary health care setting of regional and rural Victoria	To identify enablers and barriers that determine the decision of general practitioners and primary health care nurses from regional and rural Victoria to become a medication abortion provider	Quantitative (online cross-sectional survey)	Victoria	Regional areas, rural areas	General practitioners: 39<br>Primary care nurses: 30<br>Total: 69	Providers and non-providers	Male: 19<br>Female: 49<br>Unknown: 1
de Moel-Mandel 2019a[40]*	Expert consensus on a nurse-led model of medication abortion provision in regional and rural Victoria, Australia: a Delphi study	To develop a nurse-led model of medication abortion provision for the primary health care setting of regional and rural Victoria	Delphi, quantitative (online questionnaire)	Victoria	Metropolitan areas, regional areas, rural areas	Registered nurses: 10<br>Physicians: 7<br>Other professionals: 7<br>Total: 24	Providers only	Male: 4<br>Female: 19[†]
Keogh 2019a[41]	General practitioner knowledge and practice in relation to unintended pregnancy in the Grampians region of Victoria, Australia	To understand rural general practitioners' knowledge and practice in relation to unintended pregnancy and referral for abortion	Mixed methods (hardcopy surveys and semi-structured telephone interviews)	Victoria	Regional areas, rural areas	General practitioners: 23<br>Total: 23	Unknown	Male: 11<br>Female: 12

Author/Year	Title	Stated aims/objectives	Methodology	Geographical location	Participant remoteness	Participants (sample size)	Medical abortion provision status	Participant gender
Keogh 2019b[42^]	Conscientious objection to abortion, the law and its implementation in Victoria, Australia: perspectives of abortion service providers	To explore health professionals' understandings of the inclusion of Section 8 in the Abortion Law Reform Act and their perceptions of its implementation and impact on care	Qualitative (semi-structured interviews)	Victoria	Metropolitan areas, regional areas	General practitioners: 5<br>Obstetrician gynaecologists: 4<br>Medical practitioners: 3<br>Service managers: 3<br>Primary care nurses: 2<br>Psychologist: 1<br>Sexual health physician: 11<br>Total: 19	Providers and non-providers	Male: 4<br>Female: 15
Deb 2020[43]	Providing medical abortion in general practice: General practitioner insights and tips for future providers	To describe models of medical abortion care and to gain insights from current general practitioner providers about medical abortion delivery	Qualitative (semi-structured telephone interviews)	Australia	Metropolitan areas, regional areas, rural areas	General practitioners: 25<br>Total: 25	Providers only	Male: 7<br>Female: 18
de Moel-Mandel 2020[44]*	Identifying barriers and facilitators of full service nurse-led early medication abortion provision: qualitative findings from a Delphi study	To explore abortion experts' views on the factors that can influence implementation of a nurse-led model of early medical abortion provision in regional and rural primary care	Delphi, qualitative (online questionnaire)	Victoria	Metropolitan areas, regional areas, rural areas	Registered nurses: 10<br>Physicians: 7<br>Other professionals: 7<br>Total: 24	Providers only	Male: 4<br>Female: 19[†]
Ogden 2021[45]	Termination of pregnancy in Tasmania: access and service provision from the perspective of GPs**	To investigate the knowledge and attitudes of Tasmanian general practitioners regarding termination of pregnancy services, and which known barriers to providing early medical abortion are most significant	Quantitative (cross-sectional survey)	Tasmania	Metropolitan areas, rural areas	General practitioners: 211<br>Total: 211	Unknown	Male: 85<br>Female: 119<br>Unknown: 7
Mazza 2021[46]	General practitioner knowledge of and engagement with telehealth-at-home medical abortion provision	To document the experiences and opinions of general practitioners who had referred patients to a telehealth medical abortion service and their influence on access to abortion care	Qualitative (semi-structured telephone interviews)	Australia	Unknown	General practitioners: 19<br>Total: 19	Unknown	Unknown

Author/Year	Title	Stated aims/objectives	Methodology	Geographical location	Participant remoteness	Participants (sample size)	Medical abortion provision status	Participant gender
Desai 2022[47]	Views and practice of abortion among Queensland midwives and sexual health nurses	To examine the attitudes and practices of registered midwives and sexual health nurses in Queensland toward abortion	Quantitative (online cross-sectional survey)	Queensland	Metropolitan areas, regional areas, rural areas	Registered midwives: 545<br>Sexual health nurses: 78<br>Total: 623	Providers and non-providers	Male: 12<br>Female: 607<br>Other: 1<br>Unknown: 3
Malatzky 2022[48]	'I love my job…it's more the systems that we work in': the challenges encountered by rural sexual and reproductive health practitioners and implications for access to care	To examine the practice experiences and role-related challenges encountered by sexual and reproductive health practitioners practicing in three rural regions of Victoria, Australia	Qualitative (semi-structured interviews)	Victoria	Rural areas	General practitioners: 5<br>Nurses: 10<br>Total: 15	Unknown	Male: 2<br>Female: 13
Haas 2022[49]	How can we encourage the provision of early medical abortion in primary care? Results of a best-worst scaling survey	To investigate the barriers and facilitators to the provision of early medical abortion in primary care	Quantitative (online best-worst scaling survey)	Australia	Metropolitan areas, regional areas, rural areas	General practitioners: 150<br>Registered nurses: 146<br>Midwives: 4<br>Total: 300	Providers and non-providers	Male: 108<br>Female: 191<br>Other: 1
Mainey 2023[50]	Working with or against the system: Nurses' and midwives' process of providing abortion care in the context of gender- based violence in Australia	To explain the process through which Australian nurses and midwives provide abortion care to people affected by gender-based violence.	Qualitative (semi-structured interviews)	Australia	Metropolitan areas, regional areas, rural areas	Nurses and midwives: 18<br>Total: 18	Providers only	Female: 18

Author/Year	Title	Stated aims/objectives	Methodology	Geographical location	Participant remoteness	Participants (sample size)	Medical abortion provision status	Participant gender
Singh 2023[51]	General practitioner experiences in delivering early medical abortion services to women from culturally and linguistically diverse backgrounds: A qualitative-descriptive study	To explore general practitioner experiences in providing medical abortion services to women from culturally and linguistically diverse backgrounds	Qualitative (semi-structured telephone interviews)	Australia	Metropolitan areas, regional areas	General practitioners: 18<br>Total: 18	Providers only	Male: 4<br>Female: 14
Saldanha 2024[52]	Early medical abortion provision via telehealth in Victoria: A qualitative descriptive study	To understand the barriers and enablers to providing telehealth early medical abortion, as perceived by stakeholders working in this area in Victoria.	Qualitative (semi-structured interviews)	Victoria	Metropolitan, regional and rural areas	General practitioner: 6<br>Nurse/midwife: 2<br>Community SRH advocate: 2<br>Obstetrician/gynaecologist: 2<br>Sexual health physician: 1<br>Pharmacist: 1<br>Total: 14	Providers onlyAll providers	Female: 12<br>Male: 2
Sheeran 2024[53]	Attitudes towards models of abortion care in sexual and reproductive health: perspectives of Australian health professionals	To ascertain the acceptability of different models of abortion care (doctor-led, nurse-led, and self-administered) and whether attitudes differed by profession	Mixed methods (online and paper survey)	Australia	Unknown	General practitioners, obstetrician/gynaecologists:12<br>Nurses and midwives: 26<br>Administrative staff: 15<br>Total: 54	Providers onlyAll providers	Female: 47<br>Male: 5<br>Trans or non-binary: 1
Noonan 2024[54]	Imagine if we had an actual service'...': a qualitative exploration of abortion access challenges in Australian rural primary care	To describe the experiences of rural primary care providers who must negotiate the rural health system to provide local abortion services to women presenting with unintended pregnancy.	Qualitative (semi-structured interviews)	NSW	Regional and rural areas	General practitioner, general practitioner registrar, general practitioner obstetrician: 9<br>Nurse, midwife, nurse practitioner, women's health nurse: 5<br>Non-clinical: 2<br>Total: 16	Providers only	Male: 2<br>Female: 14

Author/Year	Title	Stated aims/objectives	Methodology	Geographical location	Participant remoteness	Participants (sample size)	Medical abortion provision status	Participant gender
Moulton 2024[55]	A nurse-led model of care to improve access to contraception and abortion in rural general practice: Co- design with consumers and providers	To elucidate the perspectives of consumers, health professionals and key women's health stakeholders on what constitutes an ideal nurse-led early medical abortion and long-acting reversible contraception model of care	Qualitative (online co-design workshop)	Australia	Unknown	Consumer: 5<br>Practice nurse: 3<br>Nurse practitioner: 5<br>Community/SH nurse: 7<br>General practitioner: 6<br>Academic: 4<br>Policymaker: 7<br>SRH organisation rep: 12<br>Workforce organisation rep: 3<br>Total: 52	Unknown	Unknown
Srinivasan 2024[56]	What do Australian primary care clinicians need to provide long- acting reversible contraception and early medical abortion? A content analysis of a virtual community of practice	To describe the key concerns of general practitioners, nurses and pharmacist in relation to long-acting reversible contraception and early medical abortion provision and to outline their practical and professional needs.	Mixed methods (quantitative and qualitative content analysis of online posts)	Australia	Unknown	Text-based units posted by:<br>general practitioners: 532<br>Nurses: 88<br>Pharmacists: 16<br>Total: 655 text-based units	Providers and non-providers	Unknown

*These articles report one study with qualitative and quantitative findings reported separately.

^These studies use the same participant cohort.

† These studies had missing demographic data, which is why totals do not add to 24.

4. Critical appraisal of the included studies

4.1. Qualitative studies

Study details	Screening questions		Qualitative studies questions							MMAT rating
Author	S1. Are there clear research questions ?	S2. Do the collected data allow to address the research questions ?	1.1. Is the qualitative approach appropriate to answer the research question? (Aim, appropriateness of a qualitative approach)	1.2. Are the qualitative data collection methods adequate to address the research question? (recruitment, data collection)	1.3. Are the findings adequately derived from the data? (rigor in analysis)	1.4. Is the interpretation of results sufficiently substantiated by data? (link from data to findings)	1.5. Is there coherence between qualitative data sources, collection, analysis and interpretation ? (overall design from start to finish)	1.6. Have ethical issues been taken into consideration ? (consent, confidentiality, ethics approval)	1.7. Is relationship between researcher and participants adequately considered? (interaction and reflection on how research team influences design & implementation)	
Lee 2015[34]	Yes	Yes	Yes	Yes	Unclear	Partial	Partial	Yes	No	Low (some flaws likely to impact credibility/validity)
Newton 2016[35]	Yes	Yes	Yes	Yes	Yes	Yes	Yes	Partial	No	High (no significant flaws)
Keogh 2017[36]	Yes	Yes	Yes	Yes	Yes	Yes	Yes	Yes	No	High (no significant flaws)
Dawson 2017[37]	Yes	Yes	Yes	Yes	Partial	Yes	Yes	Partial	Partial	High (no significant flaws)
Hulme-Chambers 2018[38]	Yes	Yes	Yes	Yes	Yes	Yes	Yes	Yes	Partial	High (no significant flaws)
Keogh 2019b[42]	Yes	Yes	Yes	Yes	Yes	Yes	Yes	Yes	No	High (no significant flaws)
Deb 2020[43]	Yes	Yes	Yes	Yes	Partial	Yes	Yes	Partial	No	Moderate (minor flaws impacting credibility/validity)
Mazza 2021[46]	Yes	Yes	Yes	Yes	Yes	Yes	Yes	Yes	No	High (no significant flaws)
Malatzky 2022[48]	Yes	Yes	Yes	Yes	Yes	Yes	Yes	Yes	Yes	High (no significant flaws)

Study details	Screening questions		Qualitative studies questions							MMAT rating
Author	S1. Are there clear research questions ?	S2. Do the collected data allow to address the research questions ?	1.1. Is the qualitative approach appropriate to answer the research question? (Aim, appropriatenes s of a qualitative approach)	1.2. Are the qualitative data collection methods adequate to address the research question? (recruitment , data collection)	1.3. Are the findings adequatel y derived from the data? (rigor in analysis)	1.4. Is the interpretation of results sufficiently substantiate d by data? (link from data to findings)	1.5. Is there coherence between qualitative data sources, collection, analysis and interpretation ? (overall design from start to finish)	1.6. Have ethical issues been taken into consideration ? (consent, confidentiality, ethics approval)	1.7. Is relationship between researcher and participants adequately considered? (interaction and reflection on how research team influences design & implementation)	
Mainey 2022[50]	Yes	Yes	Yes	Yes	Yes	Yes	Yes	Yes	Yes	High (no significant flaws)
Singh 2023[51]	Yes	Yes	Yes	Yes	Partial	Yes	Yes	Partial	Yes	Moderate (minor flaws impacting credibility/validity)
Saldanha 2024[52]	Yes	Yes	Yes	Yes	Yes	Yes	Yes	Partial	No	High (no significant flaws)
Noonan 2024[54]	Yes	Yes	Yes	Partial	Yes	Yes	Yes	Partial	No	High (no significant flaws)
Moulton 2024[55]	Yes	Yes	Yes	Yes	Yes	Yes	Yes	Yes	Yes	High (no significant flaws)

MMAT = Mixed Methods Appraisal Tool

4.2. Quantitative studies

Study details	Screening questions		Quantitative studies questions						MMAT rating
Author	S1. Are there clear research questions?	S2. Do the collected data allow to address the research questions?	4.1. Is the sampling strategy relevant to address the research question?	4.2. Is the sample representative of the target population?	4.3. Are the measurements appropriate?	4.4. Is the risk of nonresponse bias low?	4.5 Have ethical issues been taken into consideration? (consent, confidentiality, ethics approval)	4.6. Is the statistical analysis appropriate to answer the research question?	
de Moel-Mandel 2019b[39]	Yes	Yes	Yes	Partial	Yes	No	Yes	Yes	Moderate (minor flaws impacting credibility/validity)
Ogden 2021[45]	Yes	Yes	Yes	Partial	Yes	No	Partial	Yes	Moderate (minor flaws impacting credibility/validity)
Desai 2022[47]	Yes	Yes	Yes	Partial	Yes	No	Partial	Partial	Low (some flaws likely to impact credibility/validity)
Haas 2022[49]	Yes	Yes	Unclear	Yes	Yes	Unclear	Partial	Yes	Moderate (minor flaws impacting credibility/validity)

MMAT = Mixed Methods Appraisal Tool.

4.3. Mixed methods studies

Study details	Author and year:	Keogh 2019a[41]	Sheeran 2024[53]	Srinivasan 2024[56]
Screening questions	S1. Are there clear research questions?	Yes	Yes	Yes
	S2. Do the collected data allow to address the research questions?	Yes	Yes	Yes
1. Qualitative studies questions	1.1. Is the qualitative approach appropriate to answer the research question? (Aim, appropriateness of a qualitative approach)	Yes	Yes	Yes
	1.2. Are the qualitative data collection methods adequate to address the research question? (recruitment, data collection)	Partial	Partial	Yes
	1.3. Are the findings adequately derived from the data? (rigor in analysis)	Partial	Partial	Partial
	1.4. Is the interpretation of results sufficiently substantiated by data? (link from data to findings)	Yes	Yes	Partial
	1.5. Is there coherence between qualitative data sources, collection, analysis and interpretation? (overall design from start to finish)	Partial	Partial	Partial
	1.6. Have ethical issues been taken into consideration? (consent, confidentiality, ethics approval)	Yes	Partial	Yes
	1.7. Is relationship between researcher and participants adequately considered? (interaction and reflection on how research team influences design & implementation	No	No	Partial
2. Quantitative descriptive studies questions	4.1. Is the sampling strategy relevant to address the research question?	Yes	Yes	Yes
	4.2. Is the sample representative of the target population?	Unclear	Unclear	Unclear
	4.3. Are the measurements appropriate?	Partial	Yes	Yes
	4.4. Is the risk of nonresponse bias low?	No	No	Unclear
	4.5 Have ethical issues been taken into consideration? (consent, confidentiality, ethics approval)	Yes	Partial	Yes
	4.6. Is the statistical analysis appropriate to answer the research question?	Partial	Yes	Yes
3. Mixed methods studies questions	5.1. Is there an adequate rationale for using a mixed methods design to address the research question?	Unclear	Yes	No
	5.2. Are the different components of the study effectively integrated to answer the research question?	No	Partial	Partial
	5.3. Are the outputs of the integration of qualitative and quantitative components adequately interpreted?	No	Yes	Partial
	5.4. Are divergences and inconsistencies between quantitative and qualitative results adequately addressed?	Unclear	Unclear	Unclear
	5.5. Do the different components of the study adhere to the quality criteria of each tradition of the methods involved?	No	Partial	Partial

Study details	Author and year:	Keogh 2019a[41]	Sheeran 2024[53]	Srinivasan 2024[56]
		Very low (significant flaws impacting credibility/validity)	Low (some flaws likely to impact credibility/validity)	Low (some flaws likely to impact credibility/validity)

4.4 Delphi study

Study details	Author and year	de Moel-Mandel 2019a[40]<br>de Moel-Mandel 2020*[44]
Screening questions	S1. Are there clear research questions?	Yes
	S2. Do the collected data allow to address the research questions?	Yes
CREDES Criteria	1.1 Justification. The choice of the Delphi technique as a method of systematically collating expert consultation and building consensus needs to be well justified.	Partial
	1.2 Planning and process. The Delphi technique is a flexible method and can be adjusted to the respective research aims and purposes. Any modifications should be justified by a rationale and be applied systematically and rigorously.	Unclear
	1.3 Definition of consensus. Has consensus been defined? This includes a clear and transparent guide for action on (a) how to proceed with certain items or topics in the next survey round, (b) the required threshold to terminate the Delphi process and (c) procedures to be followed when consensus is (not) reached after one or more iterations.	Partial
	1.4 Informational input. All material provided to the expert panel at the outset of the project and throughout the Delphi process should be carefully reviewed and piloted in advance in order to examine the effect on experts' judgements and to prevent bias.	Yes
	1.5 Prevention of bias. Researchers need to take measures to avoid directly or indirectly influencing the experts' judgements. If one or more members of the research team have a conflict of interest, entrusting an independent researcher with the main coordination of the Delphi study is advisable.	Unclear
	1.6 Interpretation and processing of results. Consensus does not necessarily imply the 'correct' answer or judgement; (non)consensus and stable disagreement provide informative insights and highlight differences in perspectives concerning the topic in question.	Yes
	1.7 External validation. It is recommended to have the final draft of the resulting guidance on best practice in palliative care reviewed and approved by an external board or authority before publication and dissemination.	NA
	1.8 Purpose and rationale. The purpose of the study should be clearly defined and demonstrate the appropriateness of the use of the Delphi technique as a method to achieve the research aim. A rationale for the choice of the Delphi technique as the most suitable method needs to be provided	Partial
	1.9 Expert panel. Criteria for the selection of experts and transparent information on recruitment of the expert panel, socio-demographic details including information on expertise regarding the topic in question, (non)response and response rates over the ongoing iterations should be reported	Partial
	1.10 Description of the methods. The methods employed need to be comprehensible; this includes information on preparatory steps (How was available evidence on the topic in question synthesised?), piloting of material and survey instruments, design of the survey instrument(s), the number and design of survey rounds, methods of data analysis, processing and synthesis of experts' responses to inform the subsequent survey round and methodological decisions taken by the research team throughout the process.	Partial
CREDES criteria	1.11 Procedure. Flow chart to illustrate the stages of the Delphi process, including a preparatory phase, the actual 'Delphi rounds', interim steps of data processing and analysis, and concluding steps	Partial
	1.12 Definition and attainment of consensus. It needs to be comprehensible to the reader how consensus was achieved throughout the process, including strategies to deal with non-consensus	Partial
	1.13 Results. Reporting of results for each round separately is highly advisable in order to make the evolving of consensus over the rounds transparent. This includes figures showing the average group response, changes between rounds, as well as any modifications of the survey instrument such as deletion, addition or modification of survey items based on previous rounds	Partial

Study details	Author and year	de Moel-Mandel 2019a[40]<br>de Moel-Mandel 2020*[44]
	1.14 Discussion of limitations. Reporting should include a critical reflection of potential limitations and their impact of the resulting guidance	Partial
	1.15 Adequacy of conclusions. The conclusions should adequately reflect the outcomes of the Delphi study with a view to the scope and applicability of the resulting practice guidance	Yes
	1.16 Publication and dissemination. The resulting guidance on good practice in palliative care should be clearly identifiable from the publication, including recommendations for transfer into practice and implementation. If the publication does not allow for a detailed presentation of either the resulting practice guidance or the methodological features of the applied Delphi technique, or both, reference to a more detailed presentation elsewhere should be made (e.g. availability of the full guideline from the authors or online; publication of a separate article reporting on methodological details and particularities of the process (e.g. persistent disagreement and controversy on certain issues)). A dissemination plan should include endorsement of the guidance by professional associations and health care authorities to facilitate implementation.	NA
	Rating	Low (some flaws likely to impact credibility/validity)

***** These two articles report one study, with quantitative and qualitative results reported separately

CREDES = Guidance on Conducting and REporting DElphi Studies

5. Summary of qualitative study findings and GRADE-CERQual evidence assessments

Findings	Summary of qualitative review findings	Contributing qualitative studies	Methodological limitations	Coherence	Relevancy	Adequacy	Overall assessment	Explanation of overall assessment
**Theme 1**	**Moral, legal and regulatory influences on abortion care**							
1	Conscientious objection creates barriers to abortion care at the individual, service and system levels. Having moral or religious beliefs opposing abortion is an individual barrier to provision for some doctors and pharmacists. The presence of colleagues who conscientiously object to abortion care greatly limits opportunity for provision and clinical training. In such cases, the service (including dispensing of MS-2 Step) is not offered, care is delayed, or providers are forced to offer care clandestinely. Health services co-opt the conscientious objection legal clause to justify institutional bans on abortion provision and education.	34,36,37,41-44,46,48,50,54,56	Moderate concerns: sevenstudies with no significant issues, one study with minor issues (rigor in analysis, ethics, reflexivity), three studies with some issues (process, recruitment, rigor in analysis, link from data to findings, coherence in designs, reflexivity), one study with significant issues (recruitment, data collection, rigor in analysis, coherence in designs, reflexivity, rationale for mixed-methods approach, integration of qualitative and quantitative components)	No or very minor concerns	Moderate concerns: seven out of 12 studies directly relevant to research aim. Contributing studies were located in Victoria (five studies), NSW (three studies) and Australia-wide (4 studies). Perspectives were from general practitioners, nurses, midwives, pharmacists, clinical and non-clinical medical abortion experts and abortion service providers at multiple levels of care. Focal populations included practicing pharmacists in Sydney, NSW, and current general practitioner providers of medical abortion. Medical abortion.	No or very minor concerns: 12 studies with moderately thick data	Moderate confidence	Moderate concerns on methodological limitations (recruitment, data collection, rigor in analysis, coherence in designs, reflexivity, rationale for mixed-methods approach, integration and interpretation of qualitative and quantitative components), no or very minor concerns on coherence, moderate concerns on relevance, no or very minor concerns on adequacy (12 studies moderately thick data)
**2**	Decriminalisation is crucial but insufficient for expanding abortion care. In Victoria, decriminalisation of abortion was viewed by	36	No or very minor concerns	No or very minor concerns	Serious concerns: one article indirectly relevant to review aim. Contributing article	Serious concerns: one article contributed to this review	Very low confidence	No or very minor concerns on methodological limitations, no or very minor concerns on

Findings	Summary of qualitative review findings	Contributing qualitative studies	Methodological limitations	Coherence	Relevancy	Adequacy	Overall assessment	Explanation of overall assessment
	providers as important to symbolise that abortion care is health care. It was also understood by some as a legal mechanism to reduce unequal decisional dynamics between abortion seekers and providers of abortion care. However, in the absence of government strategies to support optimal service provision, including policies to create a sustainable health workforce, decriminalisation alone was considered insufficient to expand service provision.				represented one state (Victoria) and included both metropolitan and regional participants. Perspectives came from multiple providers at all levels of care, including general practitioners, obstetrician gynaecologists, service managers, primary health care nurses, psychologists and sexual health physicians. Focal populations are medical and surgical abortion service providers.	finding, with relatively thick data		coherence, serious concerns on adequacy (one article with relatively thick data), serious concerns on relevance (contributing article indirectly relevant to research aim)
**3**	Creating an autonomous nurse-led model of medical abortion requires regulatory reform and overcoming health system barriers. Regulations prohibiting nurse practitioner prescription of MS-2 Step, Medicare billing requirements necessitating general practitioner involvement and scarce training opportunities are systemic barriers that limit nurse scope of practice and autonomous primary care nurse provision of medical abortion. At the service level, practice nurse involvement in medical	35,44,48,52-56	Moderate concerns: five articles with no or very minor issues, three articles with some issues (process, recruitment, rigor in analysis, link from data to findings, coherence on designs, reflexivity, rationale for mixed-methods approach, integration and interpretation of qualitative and quantitative components).	No or very minor concerns	Minor concerns: five of eight articles directly relevant to review aim. Contributing articles represented Victoria (4 articles), NSW (one article) and Australia (three articles) and included participants from urban, regional and rural areas. Perspectives came from clinical and non-clinical abortion care experts (incl.	No or very minor concerns: eight articles contributed to review finding with moderately thick data	Moderate confidence	Moderate concerns on methodological limitations (process, recruitment, rigor in analysis, link from data to findings, coherence on designs, reflexivity, rationale for mixed-methods approach, integration and interpretation of qualitative and quantitative components), no or very minor

Findings	Summary of qualitative review findings	Contributing qualitative studies	Methodological limitations	Coherence	Relevancy	Adequacy	Overall assessment	Explanation of overall assessment
	abortion is dependent on employer interest and approval, and clear protocols for task sharing Conscientious objection by colleagues in regional and rural organisations may limit nurse involvement, though some have adopted alternative approaches via task-sharing with telehealth providers. Primary care nurses have communication skills well-suited to abortion care, and ease general practitioner workload when involved. However, some may lack the physical or psychological capability to independently provide abortions and manage complications without appropriate training, after-hours support and wider medical community endorsement				general practitioners, nurses, specialist physicians, academics, non-medical support staff etc.) working in a range of settings including general practice, community health, specialist and tertiary care. Focal populations are health professionals with expertise in abortion and reproductive health service provision.			concerns on coherence, minor concerns on relevance, no or very minor concerns on adequacy (eight articles with moderately thick data)
**Theme 2**	**Absence of a systems-based approach to abortion provision**							
4	There is a disconnect between primary and ancillary providers of medical abortion care. Wrap-around support from a network of ancillary services (general practitioners, pharmacists, sonographers, psychologists, referral hospitals etc.) is essential for provision of high-quality abortion care. Establishing such a network, including	35,37,38,41,43,44,48,50,52,54-56	Minor issues: eight articles with no significant issues, one article with minor issues (rigor in analysis, ethics, reflexivity), two articles with some issues (process, recruitment, rigor in analysis, link from data to findings, coherence on designs, reflexivity, rationale for mixed-	No or very minor concerns	Minor concerns: nine out of 12 studies directly relevant to review aim. Contributing articles were located in Victoria (six studies), New South Wales (two studies) and Australia-wide (4 studies), with five articles specifically rural in focus. Perspectives	No or very minor concerns: 12 articles with moderately thick data	High confidence	Minor concerns on methodological limitations (recruitment, data collection, rigor in analysis, link from data to findings, coherence in designs, reflexivity, rationale for mixed-methods approach, integration and

Findings	Summary of qualitative review findings	Contributing qualitative studies	Methodological limitations	Coherence	Relevancy	Adequacy	Overall assessment	Explanation of overall assessment
	guaranteeing the support of local hospitals for emergency and after-hours care, is logistically challenging for general practitioners given services sometimes obstruct or outright refuse to support medical abortion provision		methods approach, integration and interpretation of qualitative and quantitative components), one article with significant issues (recruitment, data collection, rigor in analysis, coherence in designs, reflexivity, rationale for mixed-methods approach, integration of qualitative and quantitative components)		came from general practitioners, nurses, midwives, abortion training providers, non-clinical medical abortion experts and abortion service providers at all levels of care. Focal populations included rural Victorian abortion training providers and their training participants.			interpretation of qualitative and quantitative components), no or very minor concerns on coherence, minor concerns on relevance, no or very minor concerns on adequacy (12 articles with moderately thick data)
**5**	Preparedness and value ascribed to training, qualifications and clinical experience. Insufficient knowledge, training and abortion care experience are barriers to in-person and telehealth abortion care provision for primary care clinicians. Many providers pursue external training or qualifications to compensate for limited opportunities provided by their workplaces, medical curricula or clinical placements. Access to supervision and hands-on learning improves general practitioners' skills and confidence, since exposure to medical abortion in primary care is often sporadic. Some experienced providers feel medical abortion care is better suited to the specialist setting where	34,35,37,48,50-52,54-56	Minor concerns: seven articles with no or very minor issues, one article with minor issues (coherence on designs), two articles with some issues (rigor in analysis, link from data to findings, coherence in designs, reflexivity, rationale for mixed-methods approach, integration and interpretation of qualitative and quantitative components)	No or very minor concerns	Minor concerns: seven out of 10 articles directly relevant to review aim. Contributing articles were located in Victoria (three articles), NSW (three articles), and Australia-wide (4 articles). perspectives were from general practitioners, nurses, midwives, pharmacists, and abortion service providers at all levels of care. focal populations included general practitioner providers of medical abortion to culturally and linguistically diverse women,	Minor concerns: 10 studies with relatively thin data	High confidence	Minor concerns on methodological limitations (coherence in designs, rigor in analysis, link from data to findings, rationale for mixed-methods approach, integration and interpretation of qualitative and quantitative components), no or very minor concerns on coherence, minor concerns on relevance, minor concerns on adequacy (10 articles relatively thin data)

Findings	Summary of qualitative review findings	Contributing qualitative studies	Methodological limitations	Coherence	Relevancy	Adequacy	Overall assessment	Explanation of overall assessment
	skills and experiences in women's health are stronger and demand for the service is consistently higher				community pharmacists and rural Victorian sexual and reproductive health workers.			
**6**	The absence of a visible service system and a culture of secrecy obscure levels of abortion demand. The absence of a visible primary care service system for abortion, particularly in rural areas, means referrers rely on "rumours" to identify primary care providers offering abortion care. Abortion stigma and privacy concerns drive practitioners to operate by 'stealth', leading to conflicting perceptions of demand – some fear being overwhelmed, while others see low demand. Abortion experts worry that without state or federal strategies for abortion service delivery, service expansion and workforce development will remain stalled.	34,36,37,41,43,46,54	Moderate concerns: 4 articles with no or very minor issues, one article with minor issues (rigor in analysis), one article with some issues (rigor in analysis, link from data to findings, coherence) and one article with significant issues (recruitment, data collection, rigor in analysis, coherence in designs, reflexivity, rationale for mixed-methods approach, integration of qualitative and quantitative components)	No or very minor concerns	Minor concerns: five out of seven studies directly relevant to review aim. Contributing articles were located in Victoria (2 articles), NSW (three articles), and Australia-wide (two articles). Perspectives came mostly from general practitioners, but also pharmacists and clinical and non-clinical abortion experts. Focal populations included general practitioners in rural Victoria, community pharmacists and current Australian general practitioner medical abortion providers.	Minor concerns: seven studies with relatively thin data	Moderate confidence	Moderate concerns on methodological limitations (recruitment, data collection, rigor in analysis, coherence in designs, reflexivity, rationale for mixed-methods approach, integration of qualitative and quantitative components), no or very minor concerns on coherence, minor concerns on relevance, minor concerns on adequacy (seven articles relatively thin data)
**7**	Under resourcing and geographic isolation are barriers to rural abortion care. Rurally based general practitioners who offer abortion care feel isolated, anticipate stigma, and experience undue pressure and emotional distress, especially when	35,37,38,41,42,44,48,50,54,55	Minor concerns: eight studies with no significant flaws, one study with some flaws (process, recruitment, link from data to findings,	No or very minor concerns	Minor concerns: seven out of 10 studies directly relevant to review aim. Contributing studies were located in Victoria (six studies), NSW (two	Minor concerns: 10 studies with moderately thin data	High confidence	Minor concerns on methodological limitations (recruitment, data collection, rigor in analysis, coherence in designs, reflexivity,

Findings	Summary of qualitative review findings	Contributing qualitative studies	Methodological limitations	Coherence	Relevancy	Adequacy	Overall assessment	Explanation of overall assessment
	working in proximity to conscientious objectors who refuse to provide clinical assistance or aid service provision. These circumstances are ignored by existing funding models, and some rural providers struggle to meet the needs of their communities without adequate resources and financial compensation leading to staff burnout, poor workforce retention and reduced quality of care for patients (e.g. delayed appointments).		coherence on designs) and one study with significant flaws (recruitment, data collection, rigor in analysis, coherence in designs, reflexivity, rationale for mixed-methods approach, integration of qualitative and quantitative components)		studies), and Australia-wide (two studies. five studies were specifically rural in focus. Perspectives were from general practitioners, nurses, midwives, abortion training providers, clinical and non-clinical abortion experts and abortion service providers working at all levels of care. focal populations included private-practice general practitioners in NSW and Australian nurses and midwives who provide abortion in the setting of gender-based violence.			rationale for mixed-methods approach, integration of qualitative and quantitative components), no or very minor concerns on coherence, minor concerns on relevance, no or very minor concerns on adequacy (10 articles moderately thin data)
**8**	Financial disincentives and the gendered nature of abortion care contribute to work overload, service fragmentation and gendered pay disparities. The fragmented structure of public healthcare financing and reliance on time-based Medicare item numbers serve as financial disincentives to provide abortion care. In the absence of a government strategy offering a supportive	36-38,43,44,46,48,51,52,54,55	Minor concerns: eight studies with no significant issues, two studies with minor flaws (rigor in analysis, coherence on designs, ethics, reflexivity) and one study with some flaws (process, recruitment, link from data to findings, coherence on designs)	No or very minor concerns	Minor concerns: eight out of 11 studies directly relevant to review aim. Contributing studies were located in Victoria (five studies), NSW (two studies) and Australia-wide (4 studies). Perspectives came from general practitioners,	No or very minor concerns: 11 studies with relatively thin data	High confidence	Minor concerns on methodological limitations (process, recruitment, link from data to findings, coherence on designs), no or very minor concerns on coherence, minor concerns on relevance, no or very minor

Findings	Summary of qualitative review findings	Contributing qualitative studies	Methodological limitations	Coherence	Relevancy	Adequacy	Overall assessment	Explanation of overall assessment
	framework for integration of abortion services into primary care, including via telehealth, individual providers bear the responsibility of navigating several logistical hurdles to facilitate access to care, doing so at a financial penalty to themselves. Coupled with the gendered nature of abortion provision and women providers often working part-time, the delays and fragmentation in care contribute to work overload and threat of burn-out at the individual-level, and gender-pay disparities and organisational glass ceilings at the service-level				nurses, abortion training providers, clinical and non-clinical medical abortion experts and abortion service providers at all levels of care. Focal populations included australian general practitioners who had referred at least one patient to a telehealth medical abortion service, and current general practitioner medical abortion providers in Australia.			concerns on adequacy (11 articles relatively thin data)
**9**	Anticipatory and enacted stigma affect abortion provision. Anticipating that medical abortion provision will detrimentally impact or otherwise subsume one's professional reputation and practice is an internalised barrier to provision for general practitioners whereas rural providers fear negative feedback from their community. Some medical abortion providers experience moral judgement from friends and colleagues and choose not to advertise their service for fear of backlash from anti-abortion activists	36,37,43,46,54,55	Minor concerns: five articles with no or very minor issues, one article with minor issues (rigor in analysis, ethics and reflexivity) and one article with some issues (process, recruitment, link from data to findings, coherence on designs),	No or very minor concerns	Minor concerns: 4 out of seven articles directly relevant to review aim. Contributing articles were located in Victoria (two articles), NSW (two articles) and Australia-wide (three articles) and six contained regional, rural or remote participants. Perspectives came from general practitioners, clinical and non-	Minor concerns: seven studies with relatively thin data	High confidence	Minor concerns on methodological limitations (process, recruitment, link from data to findings, coherence on designs, rigor in analysis, ethics and reflexivity), no or very minor concerns on coherence, minor concerns on relevance, minor concerns on adequacy (seven articles relatively thin data)

Findings	Summary of qualitative review findings	Contributing qualitative studies	Methodological limitations	Coherence	Relevancy	Adequacy	Overall assessment	Explanation of overall assessment
					clinical medical abortion experts and abortion service providers working at all levels of care. focal populations included current general practitioner providers of medical abortion and general practitioners who had referred patients to a telehealth medical abortion service			
**Theme 3**	**Early medical abortion belongs in primary care**							
10	Medical abortion in primary care enhances equity and patient autonomy. General practitioner providers are motivated by the belief that abortion care is integral to women's healthcare and should be financially, geographically and socially accessible. This sense is heightened among clinicians who provide care to socially marginalised, vulnerable or rural populations who may lack straightforward access to existing services. Medical abortion delivery in the primary setting provides greater continuity of care (e.g. for follow-up and contraception) and tailoring of care to the patient's cultural needs,	35,37,38,43,48,50-52,54	Minor concerns: seven articles with no or very minor issues, two studies with minor issues (rigor in analysis, coherence on designs, ethics and reflexivity)	No or very minor concerns	No or very minor concerns: eight out of nine studies directly relevant to review aim. Contributing studies were located in Victoria (4 studies), NSW (two studies, and Australia-wide (three studies). three studies were specifically rural in focus. Perspectives came from general practitioners, nurses, midwives, abortion training providers and abortion service providers at all levels of care. Focal populations	No or very minor concerns: nine studies with relatively thick data	High confidence	Minor concerns on methodological limitations (rigor in analysis, coherence on designs, ethics, reflexivity), no or very minor concerns on coherence, no or very minor concerns on relevance, no or very minor concerns on adequacy (nine articles relatively thick data)

Findings	Summary of qualitative review findings	Contributing qualitative studies	Methodological limitations	Coherence	Relevancy	Adequacy	Overall assessment	Explanation of overall assessment
	leading to increased provider and patient satisfaction patient and community needs including via telehealth.				included rural Victorian abortion training providers and their training participants.			

6. Summary of quantitative study findings

Findings	Summary of quantitative review findings	Contributing quantitative studies	Quality ratings
**Theme 1**	**Moral, legal and regulatory influences on abortion care**		
**1**	**Conscientious objection creates barriers to abortion care at the individual, service and system levels.** Quantitative evidence extended the qualitative finding. Some primary care providers conscientiously object to abortion however personal opposition to abortion is not always a barrier to provision of abortion care. Rates of conscientious objection appear to be higher among general practitioners trained overseas and with increasing years since qualification for registered nurses and midwives. Conscientious objection by colleagues, practice-wide bans on abortion, and pharmacist refusal to dispense MS-2 Step limit general practitioner and primary care nurse provision of abortion care.	39,40,41,45,47	five articles (two moderate, two low, one very low quality article)
**2**	**Decriminalisation is an important but insufficient mechanism to expand provision of abortion care**. This finding was supported by quantitative evidence from Queensland which suggests that decriminalisation of abortion in in the state did not significantly alter support for public provision of abortion care among sexual health nurses and midwives.	47	one low quality article
**3**	**Creating an autonomous nurse-led model of medical abortion requires regulatory reform and overcoming health system barriers.** Quantitative evidence also suggests differing views on nursing and midwifery scope of practice, concerns about handling complications, lack of abortion training opportunities, and emotional demands of abortion work, are all barriers to nurse involvement in medical abortion care. Abortion experts agree on the need for nurse-led models to expand abortion access but have conflicting views on the extent of general practitioner involvement that is necessary. Implementation of nurse-led models requires extensive government and primary health care network support to increase financial and logistical feasibility, as well as endorsement by key stakeholders such as peak nursing bodies.	39,40,47,53	four articles (one moderate, three low quality articles)
**Theme 2**	**Absence of a systems-based approach to abortion provision**		
**4**	**There is a disconnect between primary and ancillary providers of medical abortion care.** Quantitative evidence supported that inadequate access to ancillary services (pathology and ultrasound), abortion medications, and tertiary support for complications are barriers to medical abortion provision. Primary care nurses are particularly concerned about access to surgical back-up. Development of service delivery models which encompass ancillary providers and clear referral pathways is regarded as the most important factor for expanding medical abortion delivery in primary care.	39,40,49,45	Four articles (three moderate, one low quality article)
**5**	**Preparedness and value ascribed to training, qualifications and clinical experience.** Quantitative evidence supported that lack of knowledge, training opportunities, and guidelines reduce clinician preparedness. Prior experience providing abortion care is valued by general practitioners more than primary care nurses. Primary care providers desire abortion training, including as part of the core curriculum.	39,40,45,47,49,56	five articles (three moderate, three low quality articles)
**6**	**The absence of a visible service system and a culture of secrecy obscure levels of abortion demand.** Quantitative evidence supported that rurally based primary care clinicians have poor awareness of local abortion services and perceive demand to be limited due to privacy concerns. A small minority believe supply of existing abortion services is adequate. Increasing public awareness of medical abortion availability in primary care is a proposed solution to increase public demand for local abortion care, which in turn might spur provision.	39-41	Three articles (one moderate, one low, one very low quality article)
**7**	**Under resourcing and geographic isolation are barriers to rural abortion care.** Quantitative evidence supported that rural providers of medical abortion lack access to ultrasound, allied health, surgical and after-hours support. Expansion of rural medical abortion services in primary care requires further resourcing and support at the government level to incentivise training and professional development.	40	One low quality article

Findings	Summary of quantitative review findings	Contributing quantitative studies	Quality ratings
8	**Financial disincentives and the gendered nature of abortion care contribute to work overload, service fragmentation and gendered pay disparities.** Quantitative evidence supported the qualitative findings. Financial barriers to medical abortion provision include the time-cost of abortion counselling, legal restrictions preventing autonomous nurse provision, and lack of financial viability of the service. Financial considerations were more likely to be considered a significant barrier among male versus female general practitioners.	39,45,49	Three moderate quality articles
9	**Anticipatory and enacted stigma affect abortion provision.** Quantitative evidence supported that primary care clinicians, in particular nurses, are concerned about being known as abortion providers due to anticipated stigma from colleagues, friends and the wider community, and fear anti-abortion harassment.	39,40,45,40	Three articles (two moderate, one low quality article)
**Theme 3**	**Early medical abortion belongs in primary care**		
10	**Medical abortion in primary care enhances equity and patient autonomy.** *Q*uantitative evidence supported that recognition of abortion care as healthcare and the need to increase access among marginalised communities are important factors supporting provision among general practitioners and primary care nurses, however fear of loss to follow-up remains a concern.	40,47,49	Three articles (one moderate, two low quality articles)

7. References

34. Lee RY, Moles R, Chaar B. Mifepristone (RU486) in Australian pharmacies: the ethical and practical challenges. *Contraception* 2015; 91: 25-30.
35. Newton D, Bayly C, McNamee K, et al. "… a one stop shop in their own community": medical abortion and the role of general practice. *Aust N Z J Obstet Gynaecol* 2016; 56: 648-654.
36. Keogh LA, Newton D, Bayly C, et al. Intended and unintended consequences of abortion law reform: perspectives of abortion experts in Victoria, Australia. *J Fam Plann Reprod Health Care* 2017; 43: 18-24.
37. Dawson AJ, Nicolls R, Bateson D, et al. Medical termination of pregnancy in general practice in Australia: a descriptive–interpretive qualitative study. *Reprod Health* 2017; 14: 39.
38. Hulme-Chambers A, Clune S, Tomnay J. Medical termination of pregnancy service delivery in the context of decentralization: Social and structural influences. *Int J Equity Health* 2018; 17: 172.
39. de Moel-Mandel C, Graham M, Taket A. Snapshot of medication abortion provision in the primary health care setting of regional and rural Victoria. *Aust J Rural Health* 2019; 27: 237-244.
40. de Moel-Mandel C, Graham M, Taket A. Expert consensus on a nurse-led model of medication abortion provision in regional and rural Victoria, Australia: a Delphi study. *Contraception* 2019; 100: 380-385.
41. Keogh L, Croy S, Newton D, et al. General practitioner knowledge and practice in relation to unintended pregnancy in the Grampians region of Victoria, Australia. *Rural Remote Health* 2019; 19: 5156.
42. Keogh LA, Gillam L, Bismark M, et al. Conscientious objection to abortion, the law and its implementation in Victoria, Australia: perspectives of abortion service providers. *BMC Med Ethics* 2019; 20: 11.
43. Deb S, Subasinghe AK, Mazza D. Providing medical abortion in general practice: general practitioner insights and tips for future providers. *Aust J Gen Pract* 2020; 49: 331-337.
44. De Moel-Mandel C, Taket A, Graham M. Identifying barriers and facilitators of full service nurse-led early medication abortion provision: qualitative findings from a Delphi study. *Aust J Adv Nurs* 2020; 38: 18-26.
45. Ogden K, Ingram E, Levis J, et al. Termination of pregnancy in Tasmania: access and service provision from the perspective of GPs. *Aust J Prim Health* 2021; 27: 297-303.
46. Mazza D, Seymour JW, Sandhu MV, et al. General practitioner knowledge of and engagement with telehealth-at-home medical abortion provision. *Aust J Prim Health* 2021; 27: 456-461.
47. Desai A, Maier B, James-McAlpine J, et al. Views and practice of abortion among Queensland midwives and sexual health nurses. *Aust N Z J Obstet Gynaecol* 2022; 62: 219-225.
48. Malatzky C, Hulme A. "I love my job … it's more the systems that we work in": the challenges encountered by rural sexual and reproductive health practitioners and implications for access to care. *Cult Health Sex* 2022; 24: 735-749.
49. Haas M, Church J, Street DJ, et al. How can we encourage the provision of early medical abortion in primary care? Results of a best-worst scaling survey. *Aust J Prim Health* 2023; 29: 252-259.
50. Mainey L, O'Mullan C, Reid-Searl K. Working with or against the system: nurses' and midwives' process of providing abortion care in the context of gender-based violence in Australia. *J Adv Nurs* 2023; 79: 1329-1341.
51. Singh R, Mazza D, Moloney L, et al. General practitioner experiences in delivering early medical abortion services to women from culturally and linguistically diverse backgrounds: a qualitative–descriptive study. *Aust J Gen Pract* 2023; 52: 557-564.
52. Saldanha S, Caddy C, Williams H, et al. Early medical abortion provision via telehealth in Victoria: a qualitative descriptive study. *Aust J Gen Pract* 2024; 53: 794-798.
53. Sheeran N, Jones L, Corbin B, Melville C. Attitudes towards models of abortion care in sexual and reproductive health: perspectives of Australian health professionals. *Aust J Prim Health* 2024; 30: PY24100.
54. Noonan A, Millar E, Tomnay JE, et al. "Imagine if we had an actual service …": a qualitative exploration of abortion access challenges in Australian rural primary care. *Rural Remote Health* 2024; 24: 9229.
55. Moulton JE, Arefadib N, Botfield JR, et al. A nurse-led model of care to improve access to contraception and abortion in rural general practice: co-design with consumers and providers. *J Adv Nurs* 2025; 81: 1019-1037.
56. Srinivasan S, James SM, Kwek J, et al. What do Australian primary care clinicians need to provide long-acting reversible contraception and early medical abortion? A content analysis of a virtual community of practice. *BMJ Sex Reprod Health* 2025; 51: 94-101.

Preferred Reporting Items for Systematic Reviews and Meta-Analyses (PRISMA) reporting checklist

Note: The page and item numbers in this checklist refer to the submitted manuscript, not to the published article or its Supporting Information file

Section and Topic	Item #	Checklist item	Location where item is reported
Title	1	Identify the report as a systematic review.	Title
Abstract	2	See the PRISMA 2020 for Abstracts checklist.	Abstract
Rationale	3	Describe the rationale for the review in the context of existing knowledge.	Introduction
Objectives	4	Provide an explicit statement of the objective(s) or question(s) the review addresses.	Introduction (last paragraph)
Eligibility criteria	5	Specify the inclusion and exclusion criteria for the review and how studies were grouped for the syntheses.	Methods – study inclusion criteria and topic of interest, table 1
Information sources	6	Specify all databases, registers, websites, organisations, reference lists and other sources searched or consulted to identify studies. Specify the date when each source was last searched or consulted.	Methods – search methods
Search strategy	7	Present the full search strategies for all databases, registers and websites, including any filters and limits used.	Methods – Appendix S3
Selection process	8	Specify the methods used to decide whether a study met the inclusion criteria of the review, including how many reviewers screened each record and each report retrieved, whether they worked independently, and if applicable, details of automation tools used in the process.	Methods – study selection
Data collection process	9	Specify the methods used to collect data from reports, including how many reviewers collected data from each report, whether they worked independently, any processes for obtaining or confirming data from study investigators, and if applicable, details of automation tools used in the process.	Methods – data extraction and assessment of methodological limitations
Data items	10a	List and define all outcomes for which data were sought. Specify whether all results that were compatible with each outcome domain in each study were sought (e.g. for all measures, time points, analyses), and if not, the methods used to decide which results to collect.	Methods – data extraction
	10b	List and define all other variables for which data were sought (e.g. participant and intervention characteristics, funding sources). Describe any assumptions made about any missing or unclear information.	Methods – data extraction
Study risk of bias assessment	11	Specify the methods used to assess risk of bias in the included studies, including details of the tool(s) used, how many reviewers assessed each study and whether they worked independently, and if applicable, details of automation tools used in the process.	Methods – assessment of methodological limitations, Appendix S4
Effect measures	12	Specify for each outcome the effect measure(s) (e.g. risk ratio, mean difference) used in the synthesis or presentation of results.	Not applicable
Synthesis methods	13a	Describe the processes used to decide which studies were eligible for each synthesis (e.g. tabulating the study intervention characteristics and comparing against the planned groups for each synthesis (item #5)).	Not applicable
	13b	Describe any methods required to prepare the data for presentation or synthesis, such as handling of missing summary statistics, or data conversions.	Not applicable
	13c	Describe any methods used to tabulate or visually display results of individual studies and syntheses.	Methods –data analysis and synthesis
	13d	Describe any methods used to synthesize results and provide a rationale for the choice(s). If meta-analysis was performed, describe the model(s), method(s) to identify the presence and extent of statistical heterogeneity, and software package(s) used.	Methods – data analysis and synthesis

	13e	Describe any methods used to explore possible causes of heterogeneity among study results (e.g. subgroup analysis, meta-regression).	Methods – data analysis and synthesis
	13f	Describe any sensitivity analyses conducted to assess robustness of the synthesized results.	Methods – data analysis and synthesis
Reporting bias assessment	14	Describe any methods used to assess risk of bias due to missing results in a synthesis (arising from reporting biases).	Not applicable
Certainty assessment	15	Describe any methods used to assess certainty (or confidence) in the body of evidence for an outcome.	Methods – data analysis and synthesis
Study selection	16a	Describe the results of the search and selection process, from the number of records identified in the search to the number of studies included in the review, ideally using a flow diagram.	Results and Figure 1. PRISMA flowchart
	16b	Cite studies that might appear to meet the inclusion criteria, but which were excluded, and explain why they were excluded.	Figure 1. PRISMA flowchart
Study characteristics	17	Cite each included study and present its characteristics.	Results, Appendix S7
Risk of bias in studies	18	Present assessments of risk of bias for each included study.	Results and Appendix S4
Results of individual studies	19	For all outcomes, present, for each study: (a) summary statistics for each group (where appropriate) and (b) an effect estimate and its precision (e.g. confidence/credible interval), ideally using structured tables or plots.	Not applicable
Results of syntheses	20a	For each synthesis, briefly summarise the characteristics and risk of bias among contributing studies.	Results – Table 2. Summary of qualitative evidenc synthesis and Appendix S5 (GRADE CERQual evidence profile) and Appendix S7 (Summary of quantitative findings)
	20b	Present results of all statistical syntheses conducted. If meta-analysis was done, present for each the summary estimate and its precision (e.g. confidence/credible interval) and measures of statistical heterogeneity. If comparing groups, describe the direction of the effect.	Not applicable
	20c	Present results of all investigations of possible causes of heterogeneity among study results.	Not applicable
	20d	Present results of all sensitivity analyses conducted to assess the robustness of the synthesized results.	Not applicable
Reporting biases	21	Present assessments of risk of bias due to missing results (arising from reporting biases) for each synthesis assessed.	Not applicable
Certainty of evidence	22	Present assessments of certainty (or confidence) in the body of evidence for each outcome assessed.	Results – Table 2. Summary of qualitative evidenc synthesis and Appendix S5 (GRADE CERQual evidence profile) and Appendix S7 (Summary of quantitative findings)
Discussion	23a	Provide a general interpretation of the results in the context of other evidence.	Discussion
	23b	Discuss any limitations of the evidence included in the review.	Discussion – strengths and limitations
	23c	Discuss any limitations of the review processes used.	Discussion – strengths and limitations
	23d	Discuss implications of the results for practice, policy, and future research.	Discussion, conclusion

Registration and protocol	24a	Provide registration information for the review, including register name and registration number, or state that the review was not registered.	Methods
	24b	Indicate where the review protocol can be accessed, or state that a protocol was not prepared.	Methods
	24c	Describe and explain any amendments to information provided at registration or in the protocol.	Not applicable
Support	25	Describe sources of financial or non-financial support for the review, and the role of the funders or sponsors in the review.	Funding
Competing interests	26	Declare any competing interests of review authors.	Competing interests
Availability of data, code and other materials	27	Report which of the following are publicly available and where they can be found: template data collection forms; data extracted from included studies; data used for all analyses; analytic code; any other materials used in the review.	Extracted quantitative and qualitative data as well as extraction forms can be shared if requested.

Enhancing Transparency in Reporting the Synthesis of Qualitative Research: (ENTREQ) reporting checklist

Note: The page and item numbers in this checklist refer to the submitted manuscript, not to the published article or its Supporting Information file

No	Item	Guide and description	Location where item reported
1	Aim	State the research question the synthesis addresses	Introduction
2	Synthesis methodology	Identify the synthesis methodology or theoretical framework which underpins the synthesis, and describe the rationale for choice of methodology (e.g., meta-ethnography, thematic synthesis, critical interpretive synthesis, grounded theory synthesis, realist synthesis, meta-aggregation, meta-study, framework synthesis).	Methods – Data analysis and synthesis
3	Approach to searching	Indicate whether the search was pre-planned (comprehensive search strategies to seek all available studies) or iterative (to seek all available concepts until they theoretical saturation is achieved).	Methods – Search methods
4	Inclusion criteria	Specify the inclusion/exclusion criteria (e.g., in terms of population, language, year limits, type of publication, study type)	Methods – study inclusion criteria and topic of interest
5	Data sources	Describe the information sources used (e.g., electronic databases (MEDLINE, EMBASE, CINAHL, psycINFO, Econlit), grey literature databases (digital thesis, policy reports), relevant organisational websites, experts, information specialists, generic web searches (Google Scholar) hand searching, reference lists) and when the searches conducted; provide the rationale for using the data sources	Methods – Search methods
6	Electronic Search strategy	Describe the literature search (e.g., provide electronic search strategies with population terms, clinical or health topic terms, experiential or social phenomena related terms, filters for qualitative research, and search limits)	Methods – Appendix S3
7	Study screening methods	Describe the process of study screening and sifting (e.g., title, abstract and full text review, number of independent reviewers who screened studies).	Methods – study selection
8	Study characteristics	Present the characteristics of the included studies (e.g., year of publication, country, population, number of participants, data collection, methodology, analysis, research questions).	Results, Appendix S7
9	Study selection results	Identify the number of studies screened and provide reasons for study exclusion (egg, for comprehensive searching, provide numbers of studies screened and reasons for exclusion indicated in a figure/flowchart; for iterative searching describe reasons for study exclusion and inclusion based on modifications to the research question and/or contribution to theory development).	Results, Figure 1. PRISMA flowchart
10	Rationale for appraisal	Describe the rationale and approach used to appraise the included studies or selected findings (e.g., assessment of conduct (validity and robustness), assessment of reporting (transparency), assessment of content and utility of the findings).	Methods – assessment of methodological limitations, data analysis and synthesis
11	Appraisal items	State the tools, frameworks and criteria used to appraise the studies or selected findings (e.g., Existing tools: CASP, QARI, COREQ, Mays and Pope [25]; reviewer developed tools; describe the domains assessed: research team, study design, data analysis and interpretations, reporting).	Methods – assessment of methodological limitations, Appendix S4
12	Appraisal process	Indicate whether the appraisal was conducted independently by more than one reviewer and if consensus was required	Methods – assessment of methodological limitations
13	Appraisal results	Present results of the quality assessment and indicate which articles, if any, were weighted/excluded based on the assessment and give the rationale.	Appendix S4
14	Data extraction	Indicate which sections of the primary studies were analysed and how were the data extracted from the primary studies? (e.g., all text under the headings "results /conclusions" were extracted electronically and entered into a computer software).	Methods – Data extraction, data analysis and synthesis
15	Software	State the computer software used if any	Methods – data analysis and synthesis
16	Number of reviewers	Identify who was involved in coding and analysis.	Methods – data analysis and synthesis
17	Coding	Describe the process for coding of data (e.g., line by line coding to search for concepts)	Methods – Data analysis and synthesis
18	Study comparison	Describe how were comparisons made within and across studies (e.g., subsequent studies were coded into pre-existing concepts, and new concepts were created when deemed necessary)	Methods – Data analysis and synthesis

19	Derivation of themes	Explain whether the process of deriving the themes or constructs was inductive or deductive.	Methods – Data analysis and synthesis
20	Quotations	Provide quotations from the primary studies to illustrate themes/constructs and identify whether the quotations were participant quotations of the author's interpretation.	Quotations not provided as this is a qualitative evidence synthesis
21	Synthesis output	Present rich, compelling, and useful results that go beyond a summary of the primary studies (e.g., new interpretation, models of evidence, conceptual models, analytical framework, development of a new theory or construct).	Results and discussion
